# VSL#3 Co-Administration Is Associated with Altered Early-Phase Obeticholic Acid Exposure in MASLD Mice

**DOI:** 10.3390/nu18193296

**Published:** 2026-10-08

**Authors:** A-Hyeon Lee, Seung Min Lee, In Young Kang, Gye Ryeol Park, Hye Hyun Yoo, Jun Sang Yu, Hyun Sung Kim, Ji-Hee Shin, Young-Do Nam, Eileen Laurel Yoon, Dae Won Jun

**Affiliations:** 1 Center for Nucleic Acid Therapies at the Department of Chemistry Institute for Chemical Epigenetics, Ludwig-Maximilians-Universität München, Butenandtstr. 5-13, 81377 München, Germany; 2 Hanyang Institute of Bioscience and Biotechnology, Hanyang University, Seoul 04763, Republic of Korea; 3 Department of Translational Medicine, Graduate School of Biomedical Science & Engineering, Hanyang University, Seoul 04763, Republic of Korea; 4 Pharmacomicrobiomics Research Center and College of Pharmacy, Hanyang University,Ansan 15588, Republic of Korea; 5 Institute of Pharmaceutical Science and Technology and College of Pharmacy, Hanyang University, Ansan 15588, Republic of Korea; 6 College of Pharmacy, Jeju National University, Jeju 63243, Republic of Korea; 7 Department of Pathology, Hanyang University College of Medicine, Seoul 04763, Republic of Korea; 8 Research Group of Personalized Diet, Korea Food Research Institute, Wanju-gun 55365, Republic of Korea; 9 Department of Internal Medicine, Hanyang University Hospital, Hanyang University College of Medicine, 222 Wangsimni-ro, Seongdong-gu, Seoul 04763, Republic of Korea

**Keywords:** metabolic dysfunction-associated steatotic liver disease (MASLD), obeticholic acid (OCA), pharmacokinetics (PK), bile acids (BAs), microbiome

## Abstract

**Background/Objectives:** Metabolic dysfunction-associated steatotic liver disease (MASLD) is the most prevalent chronic liver disease, and its global incidence continues to increase. MASLD can progress from simple steatosis to steatohepatitis, cirrhosis, and hepatocellular carcinoma. However, effective preventive and therapeutic options remain limited. In this study, obeticholic acid (OCA) and the probiotic mixture VSL#3 were co-administered to a mouse model of MASLD to modulate gut microbiota and bile acid metabolism. **Methods:** Male C57BL/6 mice were fed a WD to induce MASLD and treated with OCA, VSL#3, or both. Gut microbiota was analyzed by 16S rRNA sequencing, bile acid (BA) composition was assessed by LC–MS/MS, hepatic and intestinal gene expression was determined by quantitative real-time PCR (qPCR) and immunohistochemistry, and unconjugated OCA pharmacokinetics (PK) were evaluated. **Results:** VSL#3-containing groups showed differences in selected taxa with reported bile salt hydrolase (BSH) potential and in BA composition, including a numerical shift toward higher levels of deconjugated BAs. Co-administration of OCA and VSL#3 was accompanied by lower hepatic steatosis, numerically favorable serum markers of liver injury, and lower cholesterol and total bilirubin levels. In addition, mice receiving VSL#3 showed numerically higher early plasma concentrations of unconjugated OCA. Lower intestinal ATP-binding cassette subfamily C member 2 (*Abcc2*) expression was also observed as a separate exploratory finding. **Conclusions:** The co-administration of VSL#3 with OCA was accompanied by exploratory differences in selected biological parameters and early-phase unconjugated OCA exposure. Lower intestinal *Abcc2*/Mrp2 expression was observed as a separate exploratory finding, and no functional relationship between intestinal transporter expression and OCA pharmacokinetics was established.

## 1. Introduction

Metabolic dysfunction-associated steatotic liver disease (MASLD) is the most prevalent chronic liver disease, and its increasing incidence imposes a substantial economic burden worldwide [1,2]. MASLD can progress to metabolic dysfunction-associated steatohepatitis (MASH), cirrhosis, and, in rare cases, hepatocellular carcinoma [3,4]. Current management of MASLD includes lifestyle modification and treatment of metabolic comorbidities; pharmacological therapies, such as resmetirom and semaglutide, are now available for selected adults with non-cirrhotic MASH and moderate-to-advanced fibrosis [5,6]. Nevertheless, effective therapeutic options remain limited for the broader MASLD population.

Recent research has highlighted the pivotal role of gut-liver axis dysfunction in the pathogenesis of liver diseases [7]. Since the liver is continuously exposed to gut-derived metabolites through the gut-liver axis, the composition and function of the gut microbiota are pivotal [8]. Accordingly, therapeutic approaches targeting the modulation of gut microbiota, such as dietary interventions and probiotics, have been proposed as adjunctive strategies to promote liver health [9]. Moreover, the gut microbiota directly influences bile acid (BA) metabolism through bile salt hydrolase (BSH) activity, thereby altering BA composition and signaling pathways that regulate hepatic metabolism and inflammation. Dysregulation of the microbiota–BA–liver axis has emerged as a key mechanism in the development and progression of MASLD.

BAs, which are synthesized from cholesterol in hepatocytes, undergo extensive metabolism and modification by microorganisms following secretion into the intestine [10]. Interactions between intestinal microbiota and BAs play a significant role in modulating host glucose homeostasis, lipid metabolism, and immune responses [10,11]. Recent studies have investigated novel therapeutic strategies targeting the gut-liver axis and BA metabolism to address the limitations of current MASLD treatment options [12,13].

Obeticholic acid (OCA), a semisynthetic BA analog and potent farnesoid X receptor (FXR) agonist, has demonstrated histological improvement, including reduction in the non-alcoholic fatty liver disease (NAFLD) Activity Score (NAS), in several clinical trials assessing its therapeutic potential for MASLD. However, the clinical response to OCA is heterogeneous, and a substantial proportion of patients show limited histological improvement or fail to achieve resolution of steatohepatitis, highlighting the need for response-optimizing strategies. Although OCA has been approved by the U.S. Food and Drug Administration for the treatment of primary biliary cholangitis, its broader application in MASLD is limited due to the adverse effects associated with long-term therapy, particularly pruritus and OCA-induced elevation in serum cholesterol levels [14,15,16]. These limitations underscore the necessity of developing combination approaches or adjunctive interventions capable of enhancing therapeutic responsiveness, while mitigating the metabolic side effects associated with OCA.

Probiotics are widely used to mitigate gastrointestinal inflammation and modulate the composition of the gut microbiota [17,18,19,20]. These therapeutic effects are mediated by both direct and indirect mechanisms, such as enhancement of epithelial barrier integrity and modulation of the immune response [9]. For example, VSL#3, a probiotic formulation used to manage ulcerative colitis, has been reported to attenuate steatohepatitis in ob/ob mice [21]. VSL#3 has been reported to upregulate genes involved in the alternative BA synthesis pathway, such as 25-hydroxycholesterol 7α-hydroxylase (*Cyp7b1*) and sterol 27-hydroxylase (*Cyp27a1*), while increasing intestinal fibroblast growth factor 15 and reducing classical BA synthesis [7]. Notably, VSL#3 contains bacterial strains for which BSH genes or activities have been reported [22,23,24].

BSH enzymes catalyze the hydrolysis of glycine- and taurine-conjugated BAs, generating unconjugated BAs, and thereby modifying the BA pool available for enterohepatic circulation. Therefore, probiotic strains with reported BSH activity may influence BA composition; however, the extent of this effect in vivo is likely to depend on strain-specific activity and the surrounding microbial community [25].

In a previous study, we demonstrated that the acidic pathway (non-12α-hydroxylated BAs) and deconjugated BAs were more abundant in the serum of OCA responders compared with non-responders prior to OCA administration [26]. Based on these findings, we hypothesized that the biological response to OCA might be influenced by VSL#3, which has been reported to modulate the pathways involved in BA metabolism and contains bacterial strains with reported BSH potential.

## 2. Materials and Methods

### 2.1. Animal

**Figure 1:**
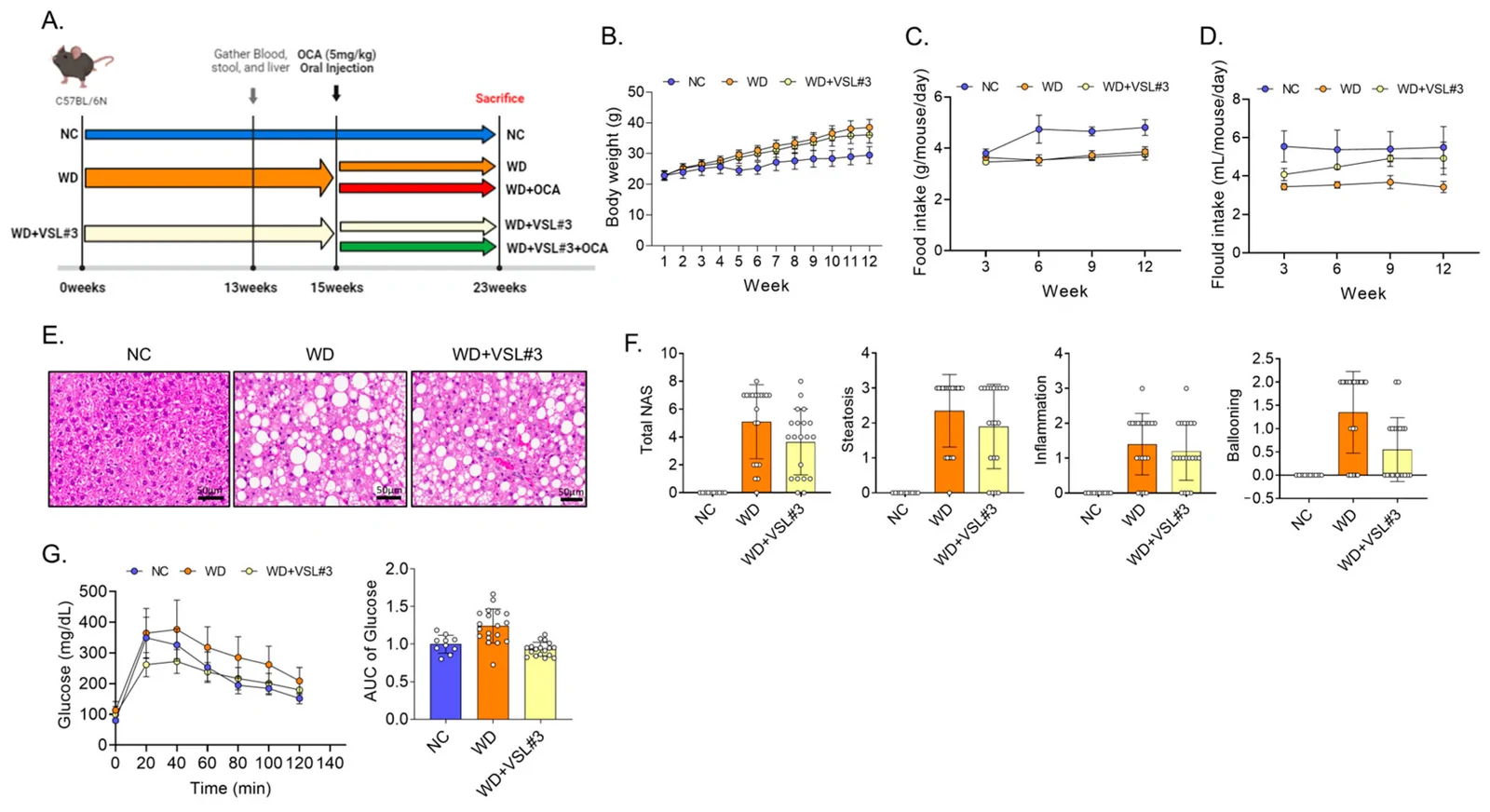
Body weight, food and fluid intake, liver histology, and glucose tolerance before OCA administration. (**A**) Schematic diagram of the experimental design. (**B**) Body weight, (**C**) food intake, and (**D**) fluid intake in mice fed a normal chow diet (NC), Western diet (WD), or WD supplemented with VSL#3. Food and fluid intake were measured at the cage level, normalized to the number of mice in each cage, and expressed as estimated daily intake per mouse. (**E**) Representative hematoxylin and eosin (H&E)-stained liver sections (100×) and (**F**) NAFLD Activity Score (NAS) at week 13. (**G**) Blood glucose concentrations during the oral glucose tolerance test (OGTT) and the corresponding area under the curve (AUC) from 0 to 120 min. Data presentation and descriptive analyses were performed as described in Section 2.15. For (**B**,**E**,**F**), n = 10 mice in the NC group, n = 20 mice in the WD group, and n = 20 mice in the WD + VSL#3 group. For (**G**), n = 10, 19, and 18 mice, respectively. For (**C**,**D**), n = 2 cages per group. Data are presented descriptively as mean ± SD with individual values where applicable. Individual cage values are shown for food and fluid intake. Because treatment allocation was performed at the cage level and only two independently allocated cages were available per experimental arm, individual mice were not considered independent experimental replicates for between-group treatment comparisons. Accordingly, no inferential statistical comparisons were used to establish treatment-group differences, and all observed group patterns are presented descriptively as exploratory biological observations.

All animal procedures were reviewed and approved by the Hanyang University Institutional Animal Care and Use Committee (approval code: HY-IACUC-24-0068; approval date: 4 August 2021) and were conducted in accordance with the approved institutional guidelines for animal care and use. C57BL/6J male mice (wild-type, 6-week-old) were obtained from the Orient Animal Laboratory (Seoul, South Korea) and randomly assigned to experimental groups. The experimental design is shown in Figure 1A. At 6 weeks of age, the animals were housed in a controlled environment (temperature, 23 ± 1–2 °C; relative humidity, 60 ± 5–10%; 12-h light/dark cycle) at the Animal Experiment Center of Hanyang University Medical School. Formal *a priori* power calculations were not performed at the time of the original study design. Eight evaluable animals per group were selected as pragmatic targets based on our preceding efficacy study using a closely matched C57BL/6J Western diet model and OCA treatment regimen [27]. A retrospective quantitative assessment performed during the present revision using endpoint NAFLD Activity Score (NAS) data from that study (vehicle vs. OCA; between-group difference, 1.07; pooled SD, 0.62), based on a two-sided two-sample *t*-test with α = 0.05, 80% power, and equal group allocation, yielded an estimated sample size of seven evaluable animals per group. Thus, the planned target of eight evaluable animals per group was consistent with this quantitative estimate. To account for anticipated biopsy-related mortality and potential sample loss, ten mice were initially allocated to each of the five experimental groups, resulting in a total of 50 mice at study initiation.

The animals were fed a normal diet, Western diet (WD), or WD supplemented with VSL#3 (purchased in August 2021) for 23 weeks, depending on the group. The OCA-treated groups received daily oral gavage of OCA (5 mg/kg body weight) following a 2-week post-operative recovery period after liver biopsy, from week 15 to week 23 (Figure 1A). In the VSL#3-containing groups, VSL#3 (9 × 10^10^ CFU/day) was administered via drinking water using the body surface area normalization method [28]. Body weight was measured individually for each mouse. Food and fluid consumption were measured at the cage level over consecutive 5-day intervals. For each cage, the total amount consumed during each 5-day interval was divided by 5 to calculate the average daily cage-level intake. This value was then normalized to the number of mice in the cage to estimate the cage-averaged daily intake per mouse. For longitudinal presentation, the estimated daily values were averaged over each 3-week interval. The cage was used as the experimental unit for the statistical analysis because food and fluid consumption were measured at the cage level. Accordingly, the exact amount of VSL#3 consumed by each mouse could not be determined. Cage-level fluid consumption was used to evaluate the variation in potential VSL#3 exposure. Since VSL#3 was supplied through drinking water shared within each cage, each cage represented an independent unit for VSL#3 administration. Individual animal outcomes were measured separately. However, mice housed within the same cage were not fully independent of VSL#3 exposure.

At the beginning of the animal experiment, the mice and their corresponding cages were randomly allocated at the cage level to the final predefined experimental groups: normal chow (NC), WD, WD + OCA, WD + VSL#3, and WD + VSL#3 + OCA. The allocation was performed immediately after arrival and before phenotypic measurements, liver biopsy, or experimental intervention. Before OCA administration, the mice assigned to the WD and WD + OCA groups received the same WD regimen, whereas the mice assigned to the WD + VSL#3 and WD + VSL#3 + OCA groups received the same WD + VSL#3 regimen. A liver biopsy was performed to evaluate the extent of the diet-induced liver phenotype before OCA administration. Following biopsy and recovery, OCA treatment was initiated only in the groups that were predefined to receive OCA. Biopsy findings were not used to assign, reassign, or modify treatment allocation.

Group allocation was not formally concealed from the investigators because the cage assignments and treatment regimens had to be managed from the beginning of the study. However, allocation was completed before any phenotypic, biochemical, or histological information was available and was not based on baseline disease severity. At the time of the liver biopsy, the histological scores showed numerical differences among the predefined treatment groups. Because treatment allocation was performed at the cage level and only two independently allocated cages were available per experimental arm, baseline histological findings were interpreted descriptively rather than as evidence of baseline equivalence. Numerical differences in baseline NAS were therefore considered when interpreting subsequent treatment-related patterns.

Three mice died from complications following the liver biopsy procedure and before OCA administration: one mouse was assigned to the WD + OCA group, and two mice were assigned to the WD + VSL#3 group. Consequently, the available post-biopsy group sizes were as follows: NC, n = 10; WD, n = 10; WD + OCA, n = 9; WD + VSL#3, n = 8; and WD + VSL#3 + OCA, n = 10. Differences in sample size across analyses were attributable to biopsy-related mortality, sample availability, insufficient sample quantity, or assay-specific analytical requirements, rather than outcome-based selective exclusion. For the microbiome analysis, all successfully collected fecal samples that yielded sufficient DNA and met the sequencing quality control criteria were included. The detailed animal numbers for each group and analysis are summarized in Supplementary Table S6.

### 2.2. Liver Biopsy and Post-Operative Care

At week 13, liver biopsy was performed to obtain liver tissue. General anesthesia was induced by intraperitoneal administration of tiletamine–zolazepam (Zoletil, 30 mg/kg) and xylazine (Rompun, 10 mg/kg). After the induction of anesthesia, a midline abdominal incision was made to expose the liver. Hemostasis was achieved by applying a heated spatula to the biopsy site, and the abdominal wall and skin were closed using 3-0 or 5-0 Vicryl sutures, as appropriate. Post-operative analgesia was provided by administering acetaminophen (paracetamol) in drinking water for 3 days. Post-operative antibiotic prophylaxis was provided by administering tetracycline (50–60 mg/L) in drinking water for 3 days. These perioperative treatments were applied identically in all experimental groups.

Following liver biopsy, the mice were monitored twice daily for 1 week for signs of post-operative complications or distress. The predefined humane endpoints included severe distress, inability to maintain a normal posture, labored breathing or unresponsiveness, and inability to access food or water. Animals meeting these criteria were euthanized by carbon dioxide inhalation, followed by cervical dislocation, in accordance with the approved institutional protocol. None of the animals underwent early euthanasia based on predefined humane endpoint criteria. The mice were allowed to recover for 2 weeks after the procedure, and the originally randomized group assignments were maintained throughout the recovery period. Subsequent interventions were initiated according to preassigned treatment groups.

### 2.3. Study Materials

Commercially available VSL#3 probiotic mixtures were purchased from TCELL BIO (Guri, Korea) and OCA was obtained from APExBIO (Boston, MA, USA).

### 2.4. Serum Biochemical Analysis

At the end of the experimental period, the healthy control and the MASLD mice were fasted for 24 h and deeply anesthetized. Terminal blood samples were collected from the abdominal aorta by exsanguination. Death was confirmed by diaphragmatic incision and opening of the thoracic cavity prior to tissue collection. The blood samples were centrifuged at 3000× *g* for 15 min at 4 °C, and the separated serum was stored at −80 °C until analysis. Serum alanine aminotransferase (ALT), aspartate aminotransferase (AST), total cholesterol, high-density lipoprotein cholesterol, low-density lipoprotein cholesterol, triglycerides, and total bilirubin levels were measured.

### 2.5. Histological Analysis

After confirming death, the livers were immediately excised and weighed to assess liver morphology. Liver tissues were fixed in 10% formalin solution, embedded in paraffin, and sectioned into 4-μm-thick slices for hematoxylin and eosin (H&E) staining. Intestinal sections were processed using the same protocol, including fixation in 10% formalin solution, paraffin embedding, and slicing into 4-μm-thick sections for H&E staining.

### 2.6. Assessment of Hepatic Steatosis, Inflammation, and Ballooning

Histological evaluation was performed based on the NAS. Hepatic steatosis, lobular inflammation, and hepatocellular ballooning were evaluated individually, and the total NAS was calculated as the sum of the three component scores. Hepatic steatosis was graded according to the percentage of the affected hepatocytes as follows: <5% (score 0), 5–33% (score 1), >33–66% (score 2), and >66% (score 3). Lobular inflammation was scored according to the number of inflammatory foci per 200× field as follows: no foci (score 0), fewer than two foci (score 1), two to four foci (score 2), and more than four foci (score 3). Hepatocellular ballooning was scored as follows: none (score 0), few ballooned hepatocytes (score 1), or many/prominent ballooned hepatocytes (score 2). Histological evaluations and NAS assessments were performed by a pathologist using coded liver sections. The pathologist was blinded to the treatment group allocation and study hypothesis during the histological assessment.

### 2.7. Quantitative Real-Time PCR (qPCR)

RNA was isolated from the liver and intestinal tissues using TRIzol reagent (Invitrogen, Carlsbad, CA, USA), following the manufacturer’s protocol. Complementary DNA (cDNA) was synthesized using a cDNA synthesis kit (Takara Bio, Shiga, Japan). qPCR was performed using SYBR Green I Master Mix (Roche Diagnostics, Indianapolis, IN, USA) on a LightCycler 480 II system. Crossing points were calculated automatically using the LightCycler software(version LCS480 1.5.1.62; Roche Diagnostics, Indianapolis, IN, USA). Each reaction was performed in duplicate or triplicate, and gene expression levels were normalized to glyceraldehyde-3-phosphate dehydrogenase (GAPDH), which served as the reference gene.

### 2.8. Western Blot Analysis

For Western blotting, the snap-frozen liver tissues were briefly rinsed in ice-cold normal saline to remove excess blood and then lysed in RIPA protein extraction solution using a tissue homogenizer. The homogenates were clarified by centrifugation, and the supernatants were collected. Protein concentration was determined with a standard protein assay, and equal amounts of protein (20 µg per lane) were mixed with sample buffer, denatured, and separated on 10% SDS-polyacrylamide gels. The proteins were transferred onto PVDF membranes (Immobilon-P; Millipore, Billerica, MA, USA). Membranes were blocked with 5% BSA and incubated with primary antibodies against cytochrome P450 family 7 subfamily A member 1 (CYP7A1), cytochrome P450 family 46 subfamily A member 1 (CYP46A1), cytochrome P450 family 27 subfamily A member 1 (CYP27A1), and GAPDH, followed by horseradish peroxidase (HRP)-conjugated secondary antibodies. Signals were developed using Amersham^TM^ ECL Prime Western blotting detection reagent (GE Healthcare, 28980926) and quantified using Image Lab software (version 5.0; Bio-Rad Laboratories, Hercules, CA, USA). CYP27A1 and CYP46A1 were selected for protein-level evaluation because both enzymes are involved in alternative cholesterol oxidation pathways related to BA and cholesterol metabolism and were assessed to determine whether transcriptional changes were also reflected at the protein level.

### 2.9. Immunohistochemical Analysis

The liver and intestinal tissues were preserved in 4% paraformaldehyde for 48 h before embedding in paraffin wax, and 4-μm-thick sections were prepared for histological staining. After paraffin removal and washing, H&E staining and immunohistochemical analyses were performed. Immunostaining was counterstained with 3,3′-diaminobenzidine, and H&E staining was counterstained with hematoxylin.

### 2.10. Intestinal Microbiome Analysis

Bacterial genomic DNA was extracted from the fecal samples using a QIAamp DNA Stool Mini Kit (Qiagen, Hilden, Germany) according to the manufacturer’s protocol. An 8-base barcoded primer was used to amplify the V1/V2 region of the 16S rRNA gene by tagging the PCR products of the samples. The amplification products were purified using the QIAquick PCR Purification Kit (Qiagen, Hilden, Germany). Subsequently, amplicons from various samples were combined in equimolar amounts. A BioAnalyzer 2100 microfluidics-based device (Agilent Technologies, Santa Clara, CA, USA) was used to assess the quality and quantity of each library constructed. Sequencing was conducted using an Ion Torrent Personal Genome Machine (Thermo Fisher Scientific, Waltham, MA, USA), according to the manufacturer’s guidelines. Raw sequencing data were processed using the QIIME 2 pipeline [29] (version 2020.11). Microbial community structure was visualized using principal coordinate analysis (PCoA) based on Bray–Curtis and Jaccard distances, and alpha diversity indices were summarized descriptively. Relative abundances of bacterial taxa were likewise summarized descriptively without inferential between-group testing. Linear discriminant analysis (LDA) effect size (LEfSe) analysis was used to generate exploratory rankings of bacterial genera using the screening criteria applied in the original analysis (LDA score of >2 and nominal *p* < 0.05; Galaxy platform, http://galaxy.biobakery.org/ (accessed on 8 September 2022)) [29]. Given the limited independent cage-level replication, the resulting LDA scores and associated screening criteria were used solely for exploratory feature ranking and were not interpreted as confirmatory evidence of treatment-related differential abundance. Basic data visualization and descriptive analyses were performed using GraphPad Prism, version 9.1.0 (GraphPad Software, Inc., San Diego, CA, USA).

### 2.11. BA Composition

Serum BA levels were analyzed at Asan Hospital, Seoul. For this analysis, approximately 30 µL of mouse serum was preprocessed according to the internal standard manual and stored at −20 °C. Before analysis, the samples were reconstituted in 50% methanol and analyzed by liquid chromatography-tandem mass spectrometry using an Agilent 1290 HPLC system (Agilent Technologies, Waldbronn, Germany) coupled with a QTRAP 5500 (AB Sciex, Toronto, ON, Canada). Mass spectrometry was conducted in the negative ion mode using multiple reaction monitoring (MRM). The chromatographic conditions were set as follows: the column temperature was maintained at 24 °C, and the flow rate was set at 200 µL/min. Quantification was performed using ion chromatograms corresponding to specific transitions of each BA. The calibration concentration range for each metabolite was from 0.1 to 10,000 nM, with an R^2^ value of ≥0.99. Data analyses were conducted using Analyst, version 1.7.1.

### 2.12. Pharmacokinetics Sample Preparation

The pharmacokinetics (PK) study was divided into two groups: a control group (WD + OCA) and a probiotic group (WD + VSL#3 + OCA). OCA (5 mg/kg) was orally administered to both groups. Blood samples were drawn from the orbital vein into heparin tubes at 0.25, 0.5, 1, 2, and 4 h after administration. The plasma was separated by centrifuging the whole blood at 13,200 rpm for 5 min and then stored at −20 °C until further analysis.

To precipitate plasma samples (20 µL), acetonitrile (40 µL) containing 10 ng/mL digitoxin (internal standard) was added. The mixture was vortexed for 1 min. The mixture was centrifuged at 13,200 rpm for 5 min. From the supernatant, 30 µL was carefully transferred to an autosampler vial, and 5 µL was injected into the LC/MS/MS system. Calibration curves were constructed for the unconjugated OCA at concentrations of 1, 3, 10, 30, 100, and 300 ng/mL. The linearity of all the calibration curves was confirmed with a correlation coefficient (R^2^) greater than 0.99. The concentrations of the calibration standards were back-calculated from the calibration curves to ensure that the calculated values did not deviate more than 15% from the theoretical values.

### 2.13. PK Analysis by LC/MS/MS

An Agilent 1290 Infinity UPLC system was paired with an Agilent 6470 triple quadrupole mass spectrometer equipped with an electrospray ionization source (Agilent Technologies, Santa Clara, CA, USA). Chromatographic separation was achieved using an ACQUITY UPLC BEH C18 column (50 × 2.1 mm, 1.7 µm; Waters), with the column oven maintained at 40 °C. HPLC separation was performed using a gradient program with mobile phase A consisting of 0.1% formic acid in distilled water and mobile phase B consisting of acetonitrile at a flow rate of 0.4 mL/min. The mobile phase gradient timetable is presented in Table 1.

The mass spectrometer was operated in the positive ion mode with electrospray ionization. Nitrogen served as the nebulizer gas at a pressure of 45 psi and as the drying gas at 300 °C and 5 L/min, and the capillary voltage was set at 3500 V. The sheath gas temperature and gas flow rate were 250 °C and 11 L/min, respectively. The fragment voltages were adjusted to 100 V and 200 V for OCA and digitoxin, respectively. MRM was performed using nitrogen as the collision gas. The Q1/Q3 ion pairs monitored were *m*/*z* 465.3 → 419.3 for OCA and *m*/*z* 763.5 → 633.0 for digitoxin.

### 2.14. PK Analysis

PK parameters were determined based on a non-compartmental approach using the Phoenix WinNonlin Enterprise Program (version 5.3; Pharsight Inc., St. Louis, MO, USA). The calculated parameters included the maximum plasma concentration (C_max_), time to maximum plasma concentration (T_max_), elimination half-life (T_1/2_), and area under the plasma concentration-time curve from time zero to the last measurable concentration (AUC_last_). Values are presented as mean ± standard deviation (SD).

### 2.15. Statistical Analysis

Unless otherwise indicated, data are presented as individual values with the mean ± SD. Descriptive data summaries and graphical presentations were prepared using GraphPad Prism 10 software (GraphPad Software, San Diego, CA, USA). Sample sizes for each analysis are provided in the corresponding figure legends and Supplementary Table S6.

Because treatment allocation for both VSL#3 and OCA was performed at the cage level, the cage represented the independent experimental unit for treatment-level inference. Individual mice housed within the same cage were therefore not considered independent experimental replicates for between-group treatment comparisons. Because only two independently allocated cages were available per experimental arm, the study did not provide sufficient independent cage-level replication for robust inferential testing of treatment effects.

Accordingly, between-group biological outcomes were summarized descriptively, and no inferential statistical testing was used to establish treatment-group differences. Individual-animal measurements are presented to transparently show the observed within-group biological distributions and variability. Body weight and other longitudinal individual-animal measurements are likewise presented descriptively. Food and fluid consumption were measured at the cage level and are presented using cage-level observations normalized to the number of mice in each cage, as described above.

Serum bile acid profiles, microbiome composition, gene-expression measurements, histological outcomes, biochemical measurements, and pharmacokinetic parameters are presented as exploratory descriptive observations. Associations between continuous biological variables were explored using Spearman’s rank correlation coefficient (ρ), which is reported descriptively without inferential *p*-values. All observed between-group patterns should therefore be considered exploratory and hypothesis-generating and require confirmation in studies with adequate independent cage-level replication.

Microbiome beta diversity was visualized using principal coordinate analysis (PCoA) based on Bray–Curtis and Jaccard distances, and alpha diversity indices were summarized descriptively. Relative abundances of selected bacterial taxa were likewise summarized descriptively without inferential between-group testing. LEfSe-derived linear discriminant analysis (LDA) effect size scores were retained solely as exploratory rankings of bacterial taxa and were not interpreted as confirmatory evidence of treatment-related differential abundance. PK parameters were summarized descriptively for the WD + OCA and WD + VSL#3 + OCA groups and were not subjected to inferential between-group testing.

## 3. Results

### 3.1. Experimental Design and Metabolic Changes Before OCA Administration

In our previous study, we found that deconjugated BAs and BAs derived from the acidic pathway were more abundant in OCA responders than in non-responders prior to OCA administration [26]. Based on these findings, VSL#3 was selected as the microbiome-targeting intervention because it has been reported to alter BA metabolism and contains bacterial strains with BSH potential.

At week 13, a liver biopsy was performed and fecal and blood samples were collected before OCA administration. Following a 2-week post-operative recovery period, the mice remained in their predefined treatment groups, and OCA was administered by daily oral gavage at 5 mg/kg/day from week 15 to week 23 (Figure 1A).

By week 13, mice fed the WD or WD supplemented with VSL#3 consumed less food and fluid than mice in the NC group, despite exhibiting increased body weight (Figure 1B–D and Supplementary Figure S1A–E). VSL#3 supplementation did not show a clear reduction in body weight or hepatic steatosis before OCA administration (Figure 1E,F). However, the blood glucose profile in the WD + VSL#3 group shifted numerically toward that observed in the NC group (Figure 1G).

### 3.2. Hepatic and Metabolic Profiles Across Experimental Groups Following OCA Treatment

**Figure 2:**
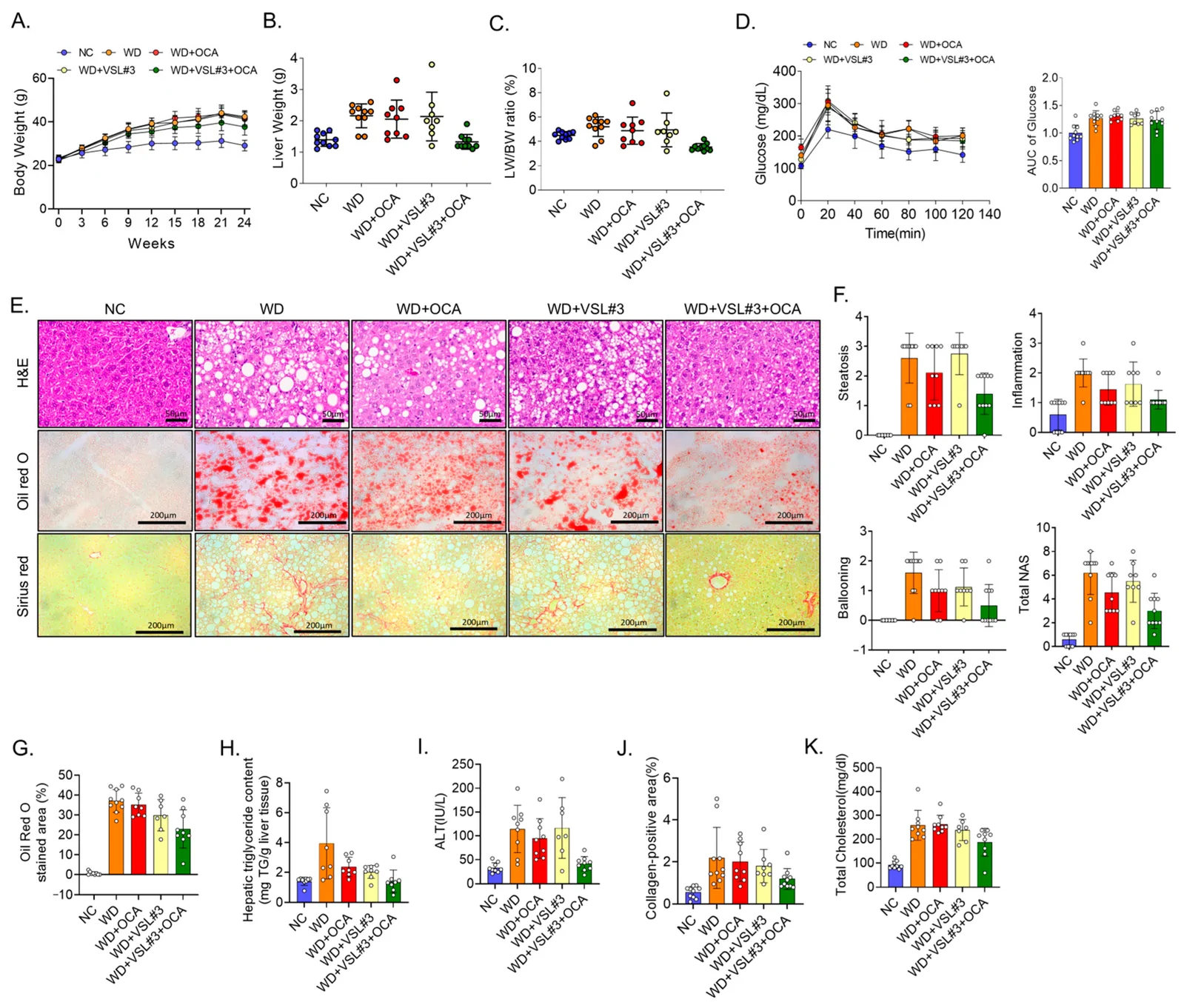
Hepatic and metabolic outcomes following co-administration of VSL#3 and Obeticholic acid (OCA). (**A**) Body weight during the 8-week OCA treatment period. (**B**) Liver weight and (**C**) liver/body weight ratio (%). (**D**) Blood glucose concentrations during the oral glucose tolerance test (OGTT) and the corresponding area under the curve (AUC) from 0 to 120 min after glucose administration. (**E**) Representative hematoxylin and eosin (H&E), Oil Red O, and Sirius Red-stained liver sections at week 23, and (**F**) NAFLD Activity Score (NAS). (**G**) Quantification of the Oil Red O-positive area, (**H**) hepatic triglyceride content, (**I**) serum alanine aminotransferase (ALT), (**J**) Sirius Red-positive area, and (**K**) serum total cholesterol. Data presentation and descriptive analyses were performed as described in Section 2.15. For (**I**,**K**), n = 9, 9, 9, 7, and 9 mice in the NC, WD, WD + OCA, WD + VSL#3, and WD + VSL#3 + OCA groups, respectively. Exact sample sizes for the remaining analyses are indicated in the figure and summarized in Supplementary Table S6. Data are presented descriptively as individual values with the mean ± SD. Because treatment allocation for both VSL#3 and OCA was performed at the cage level and only two independently allocated cages were available per experimental arm, individual mice were not considered independent experimental replicates for between-group treatment comparisons. Accordingly, no inferential statistical comparisons were used to establish treatment-group differences, and all observed group patterns are presented descriptively as exploratory biological observations.

We compared hepatic and metabolic outcomes among the WD-based treatment groups. At the end of the 8-week treatment period, the body weight remained higher in the WD-based groups than in the NC group (Figure 2A). Food intake was the highest in the NC group, whereas fluid intake was higher in the NC and WD + VSL#3 + OCA groups than in the other WD-based groups (Supplementary Figure S2A). The WD + VSL#3 + OCA group showed numerically lower liver weight and liver-to-body weight ratios, with values closer to those observed in the NC group (Figure 2B,C). Baseline liver biopsy scores showed numerical differences among the predefined treatment groups. Among all animals with available baseline histological assessments, the mean baseline NAS was lower in the WD + VSL#3 + OCA group than in the WD + OCA group (3.4 vs. 5.1, respectively; Supplementary Table S3). Since the study was not designed or powered to establish baseline equivalence, subsequent endpoint differences were interpreted in the context of these baseline imbalances. Among animals with both baseline and endpoint histological assessments, the mean NAS changed from 5.6 to 4.6 in the WD + OCA group and from 3.4 to 3.0 in the WD + VSL#3 + OCA group (Supplementary Tables S3 and S4), indicating that the numerical reduction in NAS was not greater in the combination treatment group.

Although the numerical shift in blood glucose levels observed in the WD + VSL#3 group before OCA administration was not fully maintained during continued WD feeding, the combination treatment group showed descriptive differences in several hepatic phenotypes (Figure 2D). Histological analysis showed lower hepatic steatosis in the WD + VSL#3 + OCA group compared with the WD group (Figure 2E,F).

Consistent with the histological findings, lower hepatic triglyceride accumulation and Oil Red O-positive lipid deposition were observed in the WD + VSL#3 + OCA group compared with the WD group (Figure 2E,H). Serum ALT levels in the WD + VSL#3 + OCA group were numerically closer to those observed in the NC group (Figure 2I, Supplementary Figure S2B,C).

The Sirius Red-positive area was larger in the WD group than in the NC group (Figure 2J). Numerically smaller Sirius Red-positive areas were observed in the treatment groups, including the WD + VSL#3 + OCA group (Figure 2E,J). Therefore, the fibrosis-related findings were interpreted descriptively as differences in collagen deposition rather than as evidence of fibrosis regression or an antifibrotic effect.

Serum cholesterol levels were higher in the WD + OCA group than in the WD group, whereas lower values were observed in the WD + VSL#3 + OCA group than in the WD + OCA group (Figure 2K). Serum total bilirubin and other liver-related biochemical indices also showed lower or more favorable numerical values in the combination treatment group (Supplementary Figure S3A–D). These observations showed descriptive differences in selected hepatic and metabolic parameters across the experimental groups.

### 3.3. Hepatic Expression Profiles Related to BA Synthesis and Cholesterol Handling

To examine metabolic pathways associated with the lower serum cholesterol levels observed in the combination treatment group, we assessed hepatic genes involved in BA synthesis and cholesterol transport.

Contrary to our initial expectations, numerically higher hepatic *Cyp8b1* and *Cyp7a1* expression was observed in the VSL#3-containing groups (Supplementary Figure S4A,D). *Cyp7a1* expression was numerically higher in the WD + VSL#3 + OCA group. *Cyp7b1* expression showed no clear numerical differences among the treatment groups, whereas *Cyp27a1* and *Cyp39a1* expression levels were closer to those observed in the NC group (Supplementary Figure S4B–E).

Numerically higher *Cyp46a1* expression was also observed in the WD + VSL#3 + OCA group. In addition, numerically higher *Abca1* and *Abcg1* expression was observed in the WD + VSL#3 + OCA group, whereas *Abcg5* and *Abcg8* showed similar directional patterns without clear numerical separation among the groups (Supplementary Figure S4E,F). These expression patterns were observed together with lower serum cholesterol levels in the WD + VSL#3 + OCA group than in the WD + OCA group.

### 3.4. Gut Microbiota and Circulating BA Profiles Before OCA Administration

**Figure 3:**
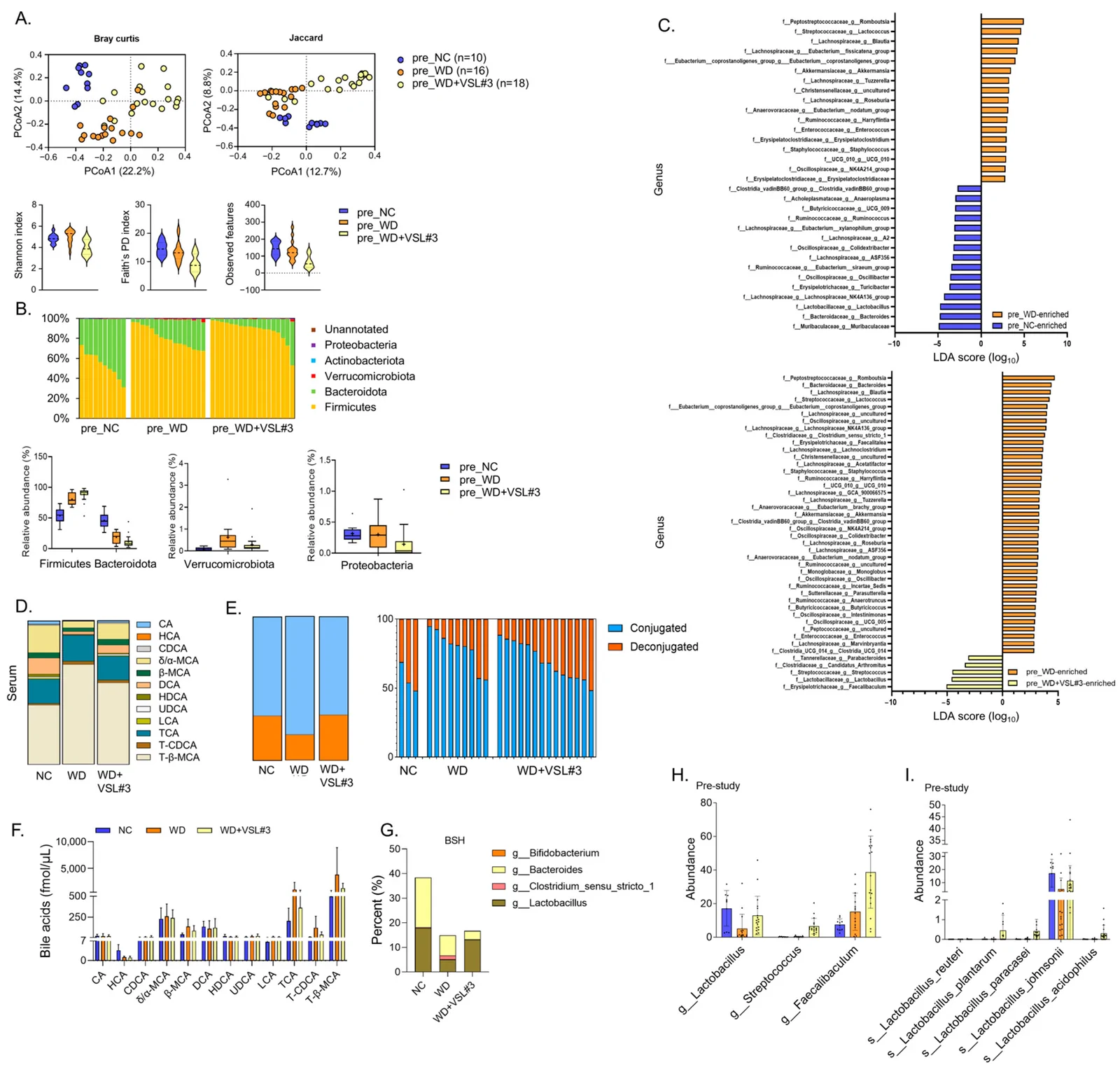
Gut microbiota and circulating bile acid profiles before OCA administration. (**A**) Beta diversity visualized using principal coordinate analysis based on Bray–Curtis and Jaccard distances (upper panel) and bacterial alpha diversity indices (lower panel) in the pre_NC, pre_WD, and pre_WD + VSL#3 groups. (**B**) Relative abundance of bacterial phyla. (**C**) Linear discriminant analysis (LDA) effect size scores generated by LEfSe. These scores are presented descriptively as an exploratory ranking of bacterial genera across the groups and are not interpreted as confirmatory evidence of treatment-related differences. For (**A**–**C**), n = 10, 16, and 18 fecal samples from the pre_NC, pre_WD, and pre_WD + VSL#3 groups, respectively. (**D**) Relative composition of individual bile acids (BAs) in serum. (**E**) Relative proportions of conjugated and deconjugated BAs. (**F**) Serum concentrations of individual BAs. (**G**) Relative abundance of putative bile salt hydrolase (BSH)-containing bacterial genera. (**H**,**I**) Relative abundances of selected bacterial taxa at the genus and species levels, respectively. For (**D**–**F**), n = 3, 9, and 14 serum samples from the NC, WD, and WD + VSL#3 groups, respectively. For (**G**–**I**), n = 10, 16, and 18 fecal samples, respectively. Detailed sample numbers are summarized in Supplementary Table S6. Data are presented descriptively as mean ± SD where applicable. Because treatment allocation was performed at the cage level and only two independently allocated cages were available per experimental arm, individual mice were not considered independent experimental replicates for between-group treatment comparisons. Accordingly, no inferential statistical comparisons were used to establish treatment-group differences, and all observed microbiome and bile acid patterns are presented as exploratory descriptive observations.

Next, we examined the microbiome and bile acid profiles preceding OCA treatment. Analysis of fecal samples collected at week 13 showed distinct gut microbial community profiles among the NC, WD, and WD + VSL#3 groups. Alpha diversity was lower in the WD + VSL#3 group than in the NC and WD groups (Figure 3A).

At the phylum level, *Firmicutes* showed higher relative abundance, *whereas Bacteroidota* showed lower relative abundance in both the WD and WD + VSL#3 groups than in the NC group. *Verrucomicrobiota* was more abundant in the WD group than in the NC group, whereas its abundance in the WD + VSL#3 group was closer to that observed in the NC group. *Proteobacteria* abundance was lower in the WD + VSL#3 group (Figure 3B).

At the genus level, exploratory LDA effect-size ranking highlighted *Romboutsia* and *Lactococcus* in the WD group and *Faecalibaculum* and *Lactobacillus* in the WD + VSL#3 group. *Lactobacillus* also showed a relatively higher abundance pattern in the NC and WD + VSL#3 groups than in the WD group (Figure 3C). These findings were interpreted descriptively as exploratory microbiome patterns.

Concurrent differences were also observed in the circulating BA profiles. Compared with the NC group, the WD group showed lower levels of δ/α-muricholic acid and deoxycholic acid and a higher level of tauro-β-muricholic acid. The WD + VSL#3 group showed δ/α-muricholic acid, deoxycholic acid, and tauro-β-muricholic acid profiles that were numerically shifted toward those observed in the NC group (Figure 3D–F).

The total abundance of taxa predicted to contain BSH did not consistently increase in the WD + VSL#3 group compared with the WD group (Figure 3G). Since BSH activity may differ among bacterial strains, we examined selected taxa with previously reported BSH potential. *Lactobacillaceae* and *Lactobacillus johnsonii* showed higher relative abundance in the WD + VSL#3 group than in the WD group (Figure 3G–I).

These exploratory findings showed concurrent differences in gut microbiome composition and circulating BA profiles. However, because the BSH gene abundance and enzymatic activity were not directly measured, the results did not demonstrate increased functional BSH activity.

### 3.5. Gut Microbiome Profiles After OCA Administration

**Figure 4:**
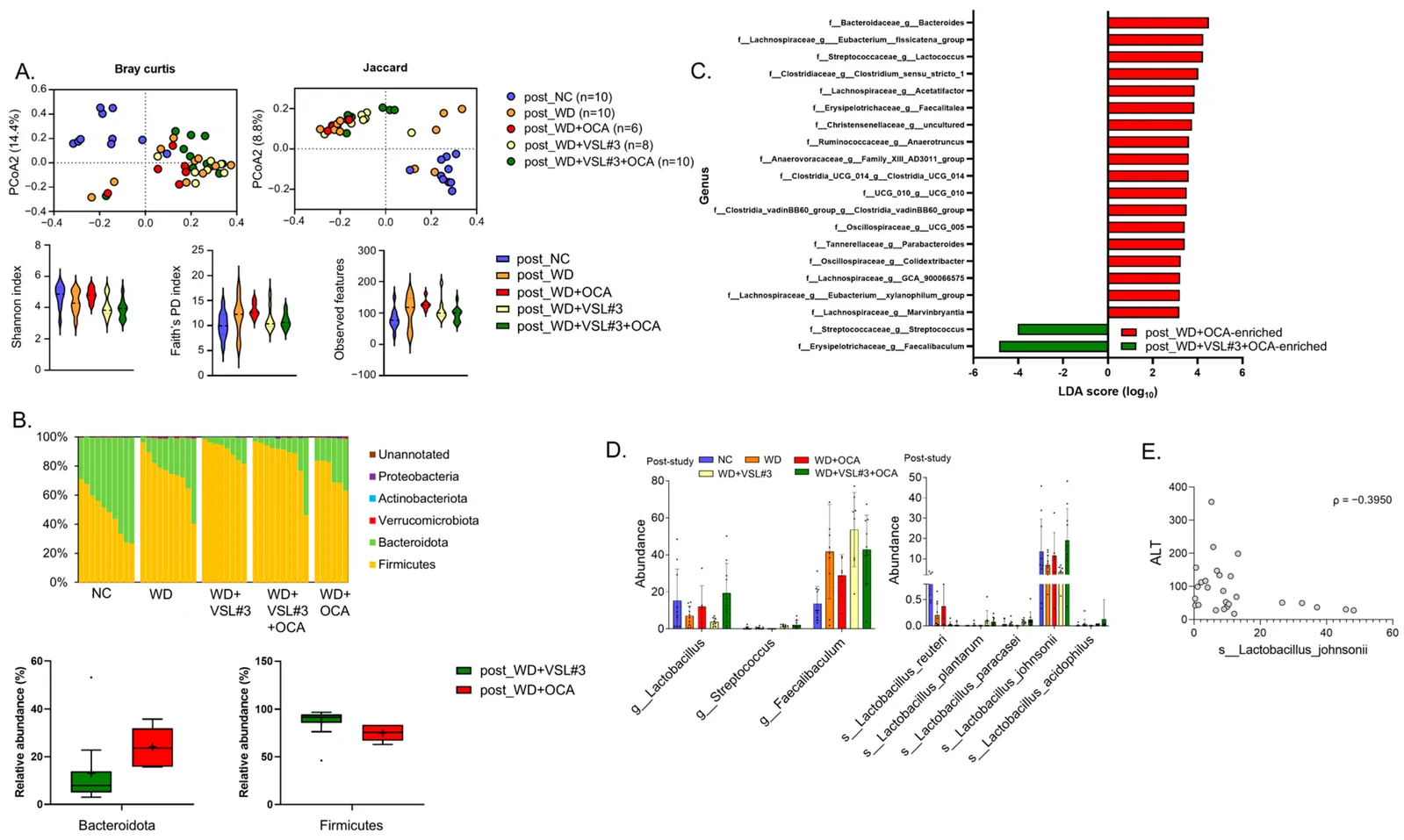
Gut microbiome composition and selected bacterial taxa after OCA administration. (**A**) Beta diversity visualized using principal coordinate analysis based on Bray–Curtis and Jaccard distances (upper panel) and bacterial alpha diversity indices (lower panel) in the five experimental groups. (**B**) Relative abundance of bacterial phyla. (**C**) Linear discriminant analysis (LDA) effect size scores generated by LEfSe. These scores are presented descriptively as an exploratory ranking of bacterial genera across the groups and are not interpreted as confirmatory evidence of treatment-related differences. For (**A**–**C**), n = 6–10 fecal samples per group. (**D**) Relative abundances of selected bacterial genera and species. (**E**) Exploratory association between the relative abundance of *Lactobacillus johnsonii* and serum ALT levels, summarized using Spearman’s rank correlation coefficient (ρ) from 27 paired samples. The correlation coefficient is reported descriptively without an inferential *p*-value. Data are presented descriptively as mean ± SD with individual values where applicable. Because treatment allocation was performed at the cage level and only two independently allocated cages were available per experimental arm, individual mice were not considered independent experimental replicates for between-group treatment comparisons. Accordingly, no inferential statistical comparisons were used to establish treatment-group differences, and all observed microbiome patterns are presented as exploratory descriptive observations.

Analysis of fecal samples collected at week 23 showed distinct gut microbial community profiles across the experimental groups, although the groups were not completely separated in the ordination plots (Figure 4A).

At the phylum level, the WD + VSL#3 + OCA group showed numerically lower *Bacteroidota* and higher *Firmicutes* abundance than the WD + OCA group. Exploratory LDA effect-size ranking also highlighted distinct genus-level microbial patterns across the treatment groups, including relatively prominent *Streptococcus* and *Faecalibaculum* profiles in the WD + VSL#3 + OCA group (Figure 4B,C). These observations were interpreted descriptively rather than as confirmatory evidence of treatment-related differential abundance.

Among the selected bacterial taxa examined descriptively, *Lactobacillus johnsonii* abundance was numerically lower in the WD group than in the NC group, numerically higher in the WD + OCA group than in the WD group, remained relatively low in the WD + VSL#3 group, and was numerically comparable to the NC group in the WD + VSL#3 + OCA group (Figure 4D).

A negative descriptive association was observed between *Lactobacillus johnsonii* abundance and serum ALT levels (Figure 4E). However, this association was exploratory and did not establish a direct causal relationship between *Lactobacillus johnsonii* abundance and liver injury-related outcomes.

### 3.6. BA Profiles, Intestinal Transporter Expression, and Early-Phase OCA Pharmacokinetics

Subsequently, we evaluated whether the microbiome profiles observed after OCA and VSL#3 administration were accompanied by differences in BA composition and intestinal transport-related pathways.

**Figure 5:**
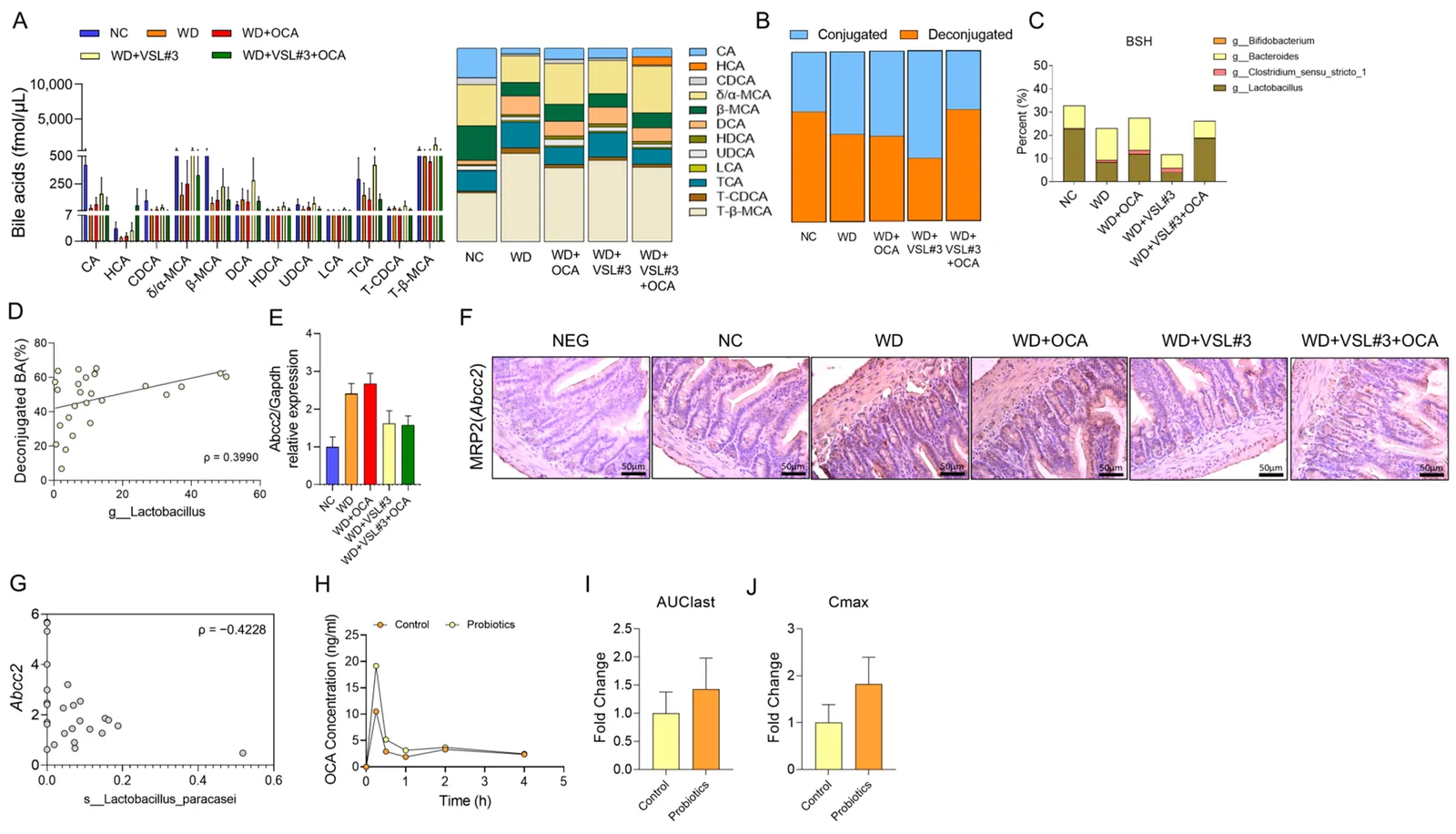
Serum bile acid profiles, intestinal transporter expression, and plasma obeticholic acid pharmacokinetics. (**A**) Serum concentrations of individual bile acids (BAs) and overall serum BA composition in the NC, WD, WD + OCA, WD + VSL#3, and WD + VSL#3 + OCA groups. Individual BA concentrations are presented descriptively without inferential between-group testing. (**B**) Proportions of conjugated and unconjugated BAs in serum. (**C**) Relative proportions of putative bile salt hydrolase (BSH)-containing bacterial taxa in fecal samples. (**D**) Exploratory association between *Lactobacillus* abundance and the proportion of unconjugated BAs, summarized using Spearman’s rank correlation coefficient (ρ) from 26 paired samples; ρ is reported descriptively, without an inferential *p*-value. For (**A**–**D**), n = 3, 4, 5, 6, and 8 in the NC, WD, WD + OCA, WD + VSL#3, and WD + VSL#3 + OCA groups, respectively. (**E**) Intestinal *Abcc2* mRNA expression; n = 10, 10, 9, 8, and 10, respectively. Values are presented descriptively without inferential between-group testing. (**F**) Representative intestinal MRP2 immunohistochemical staining (400×); n = 5 per group. NEG denotes the negative staining control. (**G**) Exploratory association between intestinal *Abcc2* mRNA expression and *Lactobacillus paracasei* abundance, summarized using Spearman’s rank correlation coefficient (ρ) from 27 paired samples; ρ is reported descriptively, without an inferential *p*-value. (**H**) Plasma OCA concentration–time profiles in the WD + OCA and WD + VSL#3 + OCA groups. (**I**,**J**) Fold changes in (**I**) AUC_last_ and (**J**) C_max_; n = 8 and 7 in the WD + OCA and WD + VSL#3 + OCA groups, respectively. Data are presented descriptively as individual biological observations with mean ± SD where applicable. Because treatment allocation was performed at the cage level and only two independently allocated cages were available per experimental arm, individual mice were not considered independent experimental replicates for between-group treatment comparisons. Accordingly, no inferential statistical comparisons were used to establish treatment-group differences. Intestinal *Abcc2*/Mrp2 expression and OCA pharmacokinetic findings were interpreted as separate exploratory observations.

Although the overall serum BA profiles showed similar trends between the two OCA-treated groups, hyocholic acid levels were numerically higher in the WD + VSL#3 + OCA group (Figure 5A). Compared with the NC group, the WD group showed lower cholic acid and chenodeoxycholic acid levels and numerically lower deconjugated BA levels. In contrast, the deconjugated BA levels in the WD + VSL#3 + OCA group were numerically higher than those in the NC group (Figure 5A,B).

Differences in deconjugated BA levels were observed together with differences in selected taxa with reported BSH potential. A positive descriptive association was observed between *Lactobacillus* abundance and deconjugated BA levels (Figure 5C,D, and Supplementary Figure S5B). *Lactobacillus johnsonii* abundance also showed negative descriptive associations with selected taurine-conjugated BAs. Nevertheless, these analyses demonstrated taxonomic and metabolic associations rather than providing direct functional evidence of microbial BA deconjugation.

Next, we examined the intestinal genes involved in BA transport, drug absorption, and efflux. Numerically lower intestinal *Abcc2* expression was observed in the VSL#3-containing groups, with a similar descriptive pattern observed at the Mrp2 protein level (Figure 5E,F, and Supplementary Figure S6A). In contrast, the mRNA levels of *Slco2b1*, *Slc51a*, *Slc51b*, *Abcc3*, and *Slc10a2* showed no clear numerical difference between the WD + OCA and WD + VSL#3 + OCA groups (Supplementary Figure S6A).

A negative descriptive association was observed between intestinal *Abcc2* expression and the relative abundance of *Lactobacillus paracasei* (Figure 5G). In addition, hyocholic acid, hyodeoxycholic acid, and δ/α-muricholic acid showed negative descriptive associations with intestinal *Abcc2*/Mrp2 expression (Supplementary Figure S6D,E). These correlations showed concurrent differences in microbiome composition, BA profiles, and intestinal *Abcc2*/Mrp2 expression, but did not establish a causal or functional relationship among these findings.

Following oral OCA administration, plasma OCA concentrations were numerically higher in the WD + VSL#3 + OCA group than in the WD + OCA group at the early sampling points (15 min to 1 h). After 2 h, plasma concentrations were broadly comparable between the groups (Figure 5H). The observed C_max_ was numerically higher in the WD + VSL#3 + OCA group, whereas AUC_last_ values were broadly comparable between the two OCA-treated groups (Figure 5I,J). T_max_ and T_1/2_ also showed no clear numerical differences between the groups (Supplementary Figure S6B,C). These descriptive PK observations were characterized primarily by higher early plasma OCA concentrations in the WD + VSL#3 + OCA group rather than by a marked difference in total systemic exposure.

Intestinal *Abcc2*/Mrp2 expression and early-phase OCA pharmacokinetics were therefore considered separate exploratory observations. The present study does not establish a functional or causal relationship between these findings.

## 4. Discussion

The gut-liver axis plays an important role in MASLD through bidirectional interactions among the gut microbiota, BA metabolism, intestinal homeostasis, and hepatic metabolic signaling [8]. BAs influence microbial composition and intestinal barrier integrity, whereas microbial enzymes modify their conjugation and composition. Disruption of this reciprocal relationship has been associated with altered intestinal permeability, BA dysregulation, and the progression of chronic liver disease [8,30]. In the present study, co-administration of VSL#3 and OCA was accompanied by descriptive differences in selected hepatic and metabolic parameters, gut microbiota composition, BA profiles, intestinal transporter expression, and early-phase OCA pharmacokinetics. Given the limited independent cage-level replication, between-group treatment findings were interpreted as exploratory biological observations rather than confirmatory treatment effects.

The VSL#3 and OCA combination group showed descriptive differences in selected hepatic and metabolic parameters, including lower hepatic steatosis, serum ALT, total bilirubin, and serum cholesterol values compared with the relevant WD-based groups. Previous clinical evidence suggested that VSL#3 supplementation may improve selected biochemical and steatosis-related parameters in patients with MASLD [31]. These exploratory observations may warrant further investigation in independently replicated studies. However, the present study was not designed to establish a formal synergistic interaction between VSL#3 and OCA.

The interpretation of histological findings should also consider the numerical imbalance in the baseline NAS between the OCA monotherapy and combination treatment groups. Because treatment allocation was performed at the cage level and independent cage-level replication was limited, baseline histological findings were interpreted descriptively rather than as evidence of baseline equivalence. Moreover, the descriptive reduction in mean NAS from baseline to the endpoint was numerically greater in the OCA monotherapy group than in the combination treatment group. Therefore, although favorable endpoint histological features were observed in the combination treatment group, the present NAS data should be interpreted cautiously and do not support attributing enhanced histological efficacy to VSL#3 co-administration.

Distinct gut microbiota composition and circulating BA profiles were observed in the VSL#3-containing groups (Figure 3). Although lower overall bacterial diversity was observed in the WD + VSL#3 group, a higher relative abundance pattern of *Lactobacillus* was observed. A positive descriptive association was observed between *Lactobacillus* abundance and deconjugated BAs. In addition, *Lactobacillus johnsonii* abundance showed negative descriptive associations with selected taurine-conjugated BAs, including tauro-chenodeoxycholic acid and tauro-β-muricholic acid (Supplementary Figure S5A,B). These findings indicated an association between selected *Lactobacillus*-related taxa and altered BA conjugation profiles.

However, these microbiome findings should not be interpreted as direct evidence of increased microbial BSH activity. Although selected *Lactobacillus*-related taxa with reported BSH potential, including *Lactobacillus johnsonii,* showed higher relative abundance in the VSL#3-containing groups, the total abundance of the predicted BSH-containing bacteria did not consistently increase (Figure 3G). Previous studies have reported that VSL#3 can increase fecal BSH activity and BA deconjugation, supporting the biological plausibility of the BA changes observed in this study [32]. However, fecal BSH activity and functional BSH gene abundance have not yet been directly assessed. Therefore, our findings indicated associations between the selected bacterial taxa and BA profiles rather than direct evidence of enhanced microbial BSH function.

We also observed an association between selected BA species and intestinal transporter expression. Hyodeoxycholic acid, hyocholic acid, and δ/α-muricholic acid levels showed negative descriptive associations with intestinal *Abcc2*/Mrp2 expression (Supplementary Figure S6D,E). These observations showed concurrent differences in selected BA species and intestinal transporter expression. However, the direction and biological basis of these relationships cannot be determined by correlation analyses alone.

A key observation was the numerically higher early plasma OCA concentration in the WD + VSL#3 + OCA group than in the WD + OCA group (Figure 5H). This descriptive pattern was characterized by a numerically higher C_max_ and higher plasma OCA concentrations at early sampling points, whereas AUC_last_ values were broadly comparable between the OCA and VSL#3 + OCA groups. These descriptive findings suggested that the observed difference in OCA exposure was more apparent during the early phase than in total systemic exposure.

However, the biological significance of this transient increase during early exposure to OCA remains unclear. Higher early plasma concentrations and C_max_ values could potentially alter the timing or magnitude of initial FXR engagement, even without a marked difference in overall systemic exposure. However, because the pharmacodynamic markers of acute FXR activation and exposure–response relationships were not assessed in the present study, it was not possible to determine whether this early PK shift contributed to the hepatic or metabolic effects observed with co-administration. From a clinical perspective, OCA treatment is associated with adverse effects, particularly dose-related pruritus and alterations in lipid profiles [33]. Nevertheless, the present findings do not establish that a transient increase in C_max_, without a marked increase in AUC_last_, enhances either therapeutic efficacy or adverse effects. Further PK–pharmacodynamic studies evaluating both peak and total OCA exposure are, therefore, required to determine the biological and clinical relevance of this early-phase PK shift.

Mrp2 is an apical ATP-binding cassette efflux transporter expressed in the intestine that is involved in the transport of BA-related compounds and xenobiotics [34,35]. In the present study, lower intestinal *Abcc2* expression was observed in the VSL#3-containing groups, with a similar pattern observed at the Mrp2 protein level. However, OCA has not been established as a direct MRP2 substrate, and the present study did not include genetic, pharmacological, or direct transporter experiments evaluating MRP2 function. Therefore, the intestinal *Abcc2*/Mrp2 findings should be regarded as exploratory observations rather than as a mechanistic explanation for the observed OCA pharmacokinetic profile.

Other intestinal processes may also be relevant to the observed early-phase OCA PK profile. Differences in the gut microbiota and BA pool among the experimental groups could potentially be associated with intestinal processing, reabsorption, or enterohepatic cycling of OCA. However, the PK analysis in the present study quantified only unconjugated OCA and did not include circulating conjugated OCA metabolites. Therefore, the observed early-phase PK changes reflected unconjugated OCA exposure and did not capture the full systemic disposition of OCA-related species. As conjugated OCA metabolites participate in enterohepatic circulation and may contribute to overall pharmacological exposure, their omission limits the interpretation of the total OCA PK profile.

This study has some limitations. First, the intestinal *Abcc2*/Mrp2 findings were exploratory and should not be interpreted as a mechanistic explanation for the observed OCA PK profile. OCA has not been established as a direct MRP2 substrate, and functional validation using Mrp2 knockdown or knockout models, pharmacological transporter inhibition, ex vivo intestinal transport assays, or intestinal epithelial cell-based OCA transport systems would be required to determine whether MRP2 contributes to OCA disposition.

Second, fibrosis-related changes were evaluated using Sirius Red staining, quantitative analysis of the Sirius Red-positive area, and hepatic expression of fibrosis-related genes. However, formal semiquantitative fibrosis staging and independent pathological grading were not performed. The Sirius Red-positive area was numerically smaller in the treatment groups than in the WD group; however, given the limited independent cage-level replication, these patterns were interpreted descriptively. Therefore, these findings should be regarded as differences in collagen deposition and fibrosis-related molecular markers, rather than as definitive evidence of fibrosis regression or an antifibrotic effect.

Third, VSL#3 was a probiotic mixture containing multiple bacterial strains, making it difficult to identify the contributions of individual microorganisms. Although *Lactobacillus johnsonii* abundance showed a negative descriptive association with serum ALT levels, this correlation did not establish a direct hepatoprotective role. Studies using isolated strains, colonization or depletion approaches, and mechanistic experimental systems are required to determine whether this species directly affects liver injury-related phenotypes.

Fourth, microbiome profiling was based on sequencing a partial region of the 16S rRNA gene, which limits species-level resolution and functional inference. Direct BSH activity measurements, targeted BSH gene quantification, and functional metagenomic analyses are required to determine whether observed taxonomic changes translate into altered microbial BA metabolism. In addition, the PK analysis quantified only unconjugated OCA, whereas circulating conjugated OCA metabolites were not measured. Consequently, the present data did not characterize the complete systemic exposure or enterohepatic disposition of OCA-related species.

An additional limitation was related to the cage-level treatment allocation and the resulting experimental-unit structure. Although OCA was administered individually by oral gavage, mice within a cage were not independently assigned to OCA versus no OCA, and VSL#3 was administered through drinking water shared within each cage. Therefore, the cage represented the independent experimental unit for treatment-level inference for both VSL#3 and OCA. Although histological, biochemical, molecular, microbiome, bile acid, and pharmacokinetic outcomes were measured in individual animals, mice housed within the same cage were not considered independent experimental replicates for between-group treatment comparisons. Because only two independently allocated cages were available per experimental arm, the study did not provide sufficient independent cage-level replication for robust inferential testing of treatment effects or VSL#3 × OCA interactions. Accordingly, all between-group treatment patterns were interpreted descriptively as exploratory and hypothesis-generating observations that require confirmation in studies with adequate independent cage-level replication.

In addition, the study was not based on a formal a priori power calculation, and the small number of independent cage-level experimental units further limited the ability to estimate treatment effects robustly.

Finally, caution is required when extrapolating these findings to human MASLD because mice and humans differ in BA composition, gut microbial ecology, intestinal transporter regulation, and enterohepatic circulation. Studies using human-derived experimental systems and prospective clinical investigations are required to determine whether the microbiome–BA–transporter associations observed in this study are relevant to human diseases.

## 5. Conclusions

In conclusion, co-administration of VSL#3 and OCA was accompanied by descriptive differences in selected hepatic, metabolic, microbiome, BA, intestinal transporter, and early-phase OCA PK profiles in WD-fed mice. Given the limited independent cage-level replication, all between-group treatment findings should be considered exploratory biological observations rather than confirmatory treatment effects. Lower intestinal *Abcc2*/Mrp2 expression and higher early plasma OCA concentrations were observed as separate findings, and no functional or causal relationship between these observations was established. Further studies with adequate independent cage-level replication and mechanistic validation are required to confirm these observations.

## Figures and Tables

**Table 1:** Gradient timetable of mobile phase.

Time (min)	Mobile Phase A (%)	Mobile Phase B (%)
0.00	70	30
0.50	40	60
1.00	40	60
1.50	30	70
2.50	30	70
2.60	2	98
3.60	2	98
3.70	70	30
6.00	70	30

## Data Availability

The gut microbiota sequencing datasets have been deposited in the Sequence Read Archive under BioProject accession number PRJNA1170011.

## Supplementary Materials

The following supporting information can be downloaded at https://www.mdpi.com/article/10.3390/nu18193296/s1: Figure S1: Metabolic and histological profiles during Western diet feeding; Figure S2: Effects of VSL#3 and OCA co-administration on food and fluid intake and hepatic inflammation- and fibrosis-related gene expression; Figure S3: Serum biochemical parameters following VSL#3 and OCA co-administration in MASLD mice; Figure S4: Hepatic expression of genes involved in bile acid synthesis and cholesterol handling following VSL#3 and OCA treatment; Figure S5: Associations between bile acid profiles and selected bacterial taxa; Figure S6: Analysis of gene expression related to intestinal drug absorption, pharmacokinetic parameters, and correlation between *Abcc2* and bile acids in MASLD mice; Table S1: Primary antibody list; Table S2: Oligonucleotide primer list for real-time PCR; Table S3: Baseline hepatic histology before OCA administration; Table S4: Endpoint hepatic histology after OCA and VSL#3 co-administration; Table S5: Summary of intestinal bile acid transport-, xenobiotic handling-, and drug metabolism-related gene expression; Table S6. Number of animals used for each analysis.

## Abbreviations

MASLD	Metabolic dysfunction-associated steatotic liver disease
NAFLD	Non-alcoholic fatty liver disease
NAS	NAFLD Activity Score
NC	Normal chow
WD	Western diet
FXR	Farnesoid X receptor
OCA	Obeticholic acid
BA	Bile acid
ALT	Alanine aminotransferase
AST	Aspartate aminotransferase
H&E	Hematoxylin and eosin
BSH	Bile salt hydrolase
LDA	Linear discriminant analysis
LEfSe	Linear discriminant analysis effect size
Mrp2	Multidrug resistance-associated protein 2
Abcc2	ATP-binding cassette subfamily C member 2
ASBT	Apical sodium-dependent bile acid transporter
OSTα/β	Organic solute transporter alpha and beta
OATP	Organic anion-transporting polypeptide
CYP46A1	Cytochrome P450 family 46 subfamily A member 1
CYP39A1	Cytochrome P450 family 39 subfamily A member 1
CYP27A1	Cytochrome P450 family 27 subfamily A member 1
CYP7B1	Cytochrome P450 family 7 subfamily B member 1
PK	Pharmacokinetics
C_max_	Maximum plasma concentration
T_max_	Time to maximum plasma concentration
T_1/2_	Elimination half-life
AUC_last_	Area under the plasma concentration–time curve from time zero to the last measurable concentration
PCoA	Principal coordinate analysis
CA	Cholic acid
CDCA	Chenodeoxycholic acid
T-CDCA	Tauro-chenodeoxycholic acid
α-MCA	α-muricholic acid
T-β-MCA	Tauro-β-muricholic acid
DCA	Deoxycholic acid
HCA	Hyocholic acid
HDCA	Hyodeoxycholic acid
