# Supplementary material for: VSL#3 Co-Administration Is Associated with Altered Early-Phase Obeticholic Acid Exposure in MASLD Mice
Source: Nutrients. 2026 Oct 8;18(19):3296. doi: 10.3390/nu18193296 (PMC13667209; doi:10.3390/nu18193296)

Supplementary Information

VSL#3 Co-Administration Is Associated with Altered Early-Phase Obeticholic Acid Exposure in MASLD Mice

A-Hyeon Lee [1,†], Seung Min Lee [2,3,†], In Young Kang [3], Gye Ryeol Park [3], Hye Hyun Yoo [4], Jun Sang Yu [5,6], Hyun Sung Kim [7], Ji-Hee Shin [8], Young-Do Nam [8], Eileen Laurel Yoon [3,9,‡] and Dae Won Jun [3,9,*]

Table of contents………………………………………………………..……..……… 1– 2

Supplementary Tables S1 – S6 ……………………………………………….……... 3–14

Supplementary Table S1. Primary antibody list …………………..………..……..………... 3

Supplementary Table S2. Oligonucleotide primer list for real-time PCR………….........…4–5

Supplementary Table S3. Baseline hepatic histology before OCA administration………….. 6–8

Supplementary Table S4. Endpoint hepatic histology after OCA and VSL#3 co-administration…………………………………………………………………….…... 9–11

Supplementary Table S5. Summary of intestinal bile acid transport-, xenobiotic handling-, and drug metabolism-related gene expression..…………………..…………….……….….… 12–13

Supplementary Table S6. Number of animals used for each analysis…………………...…..14–15

Supplementary Figures and Legends S1–S6……………………………………..….. 16 – 21

Supplementary Figure S1. Metabolic and histological profiles during Western diet feeding… 16

Supplementary Figure S2. Effects of VSL#3 and OCA co-administration on food and fluid intake

and hepatic inflammation- and fibrosis-related gene expression …………….……………………..17

Supplementary Figure S3. Serum biochemical parameters following VSL#3 and OCA co-administration in MASLD mice...…………………………………………..…………………….... 18

Supplementary Figure S4. Hepatic expression of genes involved in bile acid synthesis and cholesterol handling following VSL#3 and OCA treatment.. 19

Supplementary Figure S5. Associations between bile acid profiles and selected bacterial taxa... 20

Supplementary Figure S6. Analysis of gene expression related to intestinal drug absorption, pharmacokinetic parameters, and correlation between Abcc2 and bile acids in MASLD mice.…………………………..…………………………………………………….………… 21

Supplementary table

Supplementary table S1. Primary antibody list.

**Target**	**Host/ Clonality**	**Company/ Product number**	Application **Dilution**	**Validation details**
MRP2	Rabbit/ Polyclonal	Bioss, bs-1092R	IHC-P 1:150	Manufacturer-documented for IHC-P using formalin-fixed, paraffin-embedded human liver carcinoma tissue at 1:200 with DAB-based detection. In this study, the antibody was applied to formalin-fixed, paraffin-embedded mouse intestinal sections under optimized IHC-P conditions.
CYP7A1	Rabbit/ Polyclonal	Bioss, bs-2399R	WB 1:1000	Manufacturer-listed for WB, ELISA, IHC-P, IF(IHC-F), and IF(ICC). The antibody is reported as reactive with human and guinea pig, with predicted reactivity in mouse, rat, pig, and rabbit. In this study, the antibody was used under optimized conditions for mouse samples.
CYP46A1	Rabbit/ Polyclonal	Abcam, ab82814	WB 1:1000	Manufacturer-documented for WB, with predicted molecular weight of approximately 57 kDa. The datasheet lists human reactivity and predicted reactivity with mouse and other species. In this study, the antibody was applied to mouse liver lysates, and western blot detection was interpreted based on the expected molecular weight.
CYP27A1	Rabbit/ Polyclonal	Bioss, bs-5049R	WB 1:1000	The manufacturer lists this antibody for WB, ELISA, IHC-P, IHC-F, IF(IHC-P), IF(IHC-F), and IF(ICC), and reports mouse reactivity. Mouse sample application in this study was based on manufacturer-reported mouse reactivity and optimized experimental conditions.
GAPDH	Rabbit/ Polyclonal	GeneTex, GTX100118	WB 1:1000	Manufacturer-validated for WB, ICC/IF, IHC-P, and IP, with broad species reactivity including human, mouse, and rat. In this study, GAPDH was used as a loading control for western blot analysis.

Supplementary table S2. Oligonucleotide primer list for real-time PCR.

Gene	Forward Primer Sequences (5' – 3')	Reverse Primer Sequences (5' – 3')
Cyp39a1	TCATTCTGGAACCCTCCTGC	CAGCCCAAAGTACGACCAGT
Cyp46a1	CATGAGACTTCTGCCAACCA	CTTGGAACCGACAACCTCAT
Cyp7a1	CAACGGGTTGATTCCATACC	ATTTCCCCATCAGTTTGCAG
Cyp7b1	TTCTCTGGGCCTCTCTAGCA	CAGGGCTTCCATAGCTTCAG
Cyp8b1	TGGCCTCTTTCACTTCTGCT	CGGAACTTCCTGAACAGCTC
Gapdh	GTTGTCTCCTGCGACTTC	GGTGGTCCAGGGTTTCTT
Abca1	GGAGCCTTTGTGGAACTCTTCC	CGCTCTCTTCAGCCACTTTGAG
Abcg1	GACACCGATGTGAACCCGTTTC	GCATGATGCTGAGGAAGGTCCT
Abcg5	TGCCATCCTGACTTACGGAGAG	CTGCTTTGGGTGTCCACTGATG
Abcg8	GGTCCTTCTGATGACATCTGGC	CGTCTGTCGATGCTGGTCAAGT
Cdh17	GTCACAGATCCCACGACCAATG	CCTTTCACCAGTGGCTCAGGAT
Abcb1b	GGTGGTGTCATTGTGGAGCAAG	GCATCAGTGTCACTCTGGGATC
Abcc2	TACCAGCGAGTTATCGAAGCGTG	TGCTTCTGACCGCCACTGAGAT
Abcc3	ACTTCCTCCGAAACTACGCACC	GCTGGCTCATTGTCTGTCAGGT
Cyp2b10	AAGGAGAAGTCCAACCAGCA	CTCTGCAACATGGGGGTACT
Slco2b1	TCAGGACTCACATCAGGATGC	CTCTTGAGGTAGCCAGAGATCA
Slc51b	GAGAAACCAGACTTGGCCCC	TCTCAACGCTCCCTAGCTCT
Slc51a	TACAAACACCCTTTGCCC	CGAGGAATCCAGAGACCAAA
Asbt	GACTCGGGAACGATTGTGAT	GGTTCAATGATCCAGGCACT
Col1a1	CCTCAGGGTATTGCTGGACAAC	CAGAAGGACCTTGTTTGCCAGG

Tgfβ1	GACTCTCCACCTGCAAGACC	GACTGGCGAGCCTTAGTTTG
Tnfα	GGTGCCTATGTCTCAGCCTCTT	GCCATAGAACTGATGAGAGGGAG
Ccl2	GCTACAAGAGGATCACCAGCAG	GTCTGGACCCATTCCTTCTTGG
Pck1	GGCGATGACATTGCCTGGATGA	TGTCTTCACTGAGGTGCCAGGA
G6pc	AGGTCGTGGCTGGAGTCTTGTC	GTAGCAGGTAGAATCCAAGCGC
Foxo1	CTACGAGTGGATGGTGAAGAGC	CCAGTTCCTTCATTCTGCACTCG
Irs2	CCAGTAAACGGAGGTGGCTACA	CCATAGACAGCTTGGAGCCACA
Slc2a2	GTTGGAAGAGGAAGTCAGGGCA	ATCACGGAGACCTTCTGCTCAG

Supplementary table S3. Baseline hepatic histology before OCA administration

Group	Steatosis	Lobular inflammation	Ballooning	Total NAS
NC1	0	0	0	0
NC2	0	0	0	0
NC3	0	0	0	0
NC4	0	0	0	0
NC5	0	0	0	0
NC6	0	0	0	0
NC7	0	0	0	0
NC8	0	0	0	0
NC9	0	0	0	0
NC10	0	0	0	0
WD1	3	2	2	7
WD2	3	2	2	7
WD3	3	2	2	7
WD4	3	1	1	5
WD5	3	2	2	7
WD6	1	1	0	2
WD7	1	1	0	2
WD8	3	2	2	7
WD9	0	0	0	0
WD10	3	2	2	7

WD+OCA1	3	3	2	8
WD+OCA2	3	1	2	6
WD+OCA3	3	2	2	7
WD+OCA4	3	2	2	7
WD+OCA5	3	2	2	7
WD+OCA6	3	1	1	5
WD+OCA7	3	2	2	7
WD+OCA8	1	0	0	1
WD+OCA9	1	0	1	2
WD+OCA10	1	0	0	1
WD+VSL#3 1	3	2	2	7
WD+VSL#3 2	0	1	0	1
WD+VSL#3 3	0	0	0	0
WD+VSL#3 4	3	2	1	6
WD+VSL#3 5	3	1	1	5
WD+VSL#3 6	2	1	1	4
WD+VSL#3 7	3	1	0	4
WD+VSL#3 8	3	1	1	5
WD+VSL#3 9	1	1	0	2
WD+VSL#3 10	3	2	0	5
WD+VSL#3+OCA 1	0	1	0	1
WD+VSL#3+OCA 2	2	2	0	4
WD+VSL#3+OCA 3	2	1	1	4

WD+VSL#3+OCA 4	3	3	2	8
WD+VSL#3+OCA 5	1	0	0	1
WD+VSL#3+OCA 6	2	2	0	4
WD+VSL#3+OCA 7	1	0	0	1
WD+VSL#3+OCA 8	0	0	0	0
WD+VSL#3+OCA 9	3	2	1	6
WD+VSL#3+OCA 10	3	1	1	5

Supplementary table S4. Endpoint hepatic histology after OCA and VSL#3 co-administration

**Group**	**Steatosis**	**Lobular inflammation**	**Ballooning**	**Total NAS**
NC1	0	1	0	1
NC2	0	0	0	0
NC3	0	0	0	0
NC4	0	0	0	0
NC5	0	1	0	1
NC6	0	1	0	1
NC7	0	1	0	1
NC8	0	1	0	1
NC9	0	0	0	0
NC10	0	1	0	1
WD1	3	2	2	7
WD2	3	2	1	6
WD3	3	2	2	7
WD4	3	2	2	7
WD5	3	2	2	7
WD6	1	2	1	4
WD7	3	2	2	7
WD8	3	3	2	8
WD9	1	1	0	2
WD10	3	2	2	7

WD+OCA1	3	2	1	6
WD+OCA2	1	2	0	3
WD+OCA3	3	2	2	7
WD+OCA4	1	1	1	3
WD+OCA5	3	2	1	6
WD+OCA6	2	1	0	3
WD+OCA7	3	1	2	6
WD+OCA8	2	1	1	4
WD+OCA9	1	1	1	3
WD+VSL#3 1	3	1	1	5
WD+VSL#3 2	3	2	1	6
WD+VSL#3 3	1	1	0	2
WD+VSL#3 4	3	3	2	8
WD+VSL#3 5	3	2	2	7
WD+VSL#3 6	3	1	1	5
WD+VSL#3 7	3	2	1	6
WD+VSL#3 8	3	1	1	5
WD+VSL#3+OCA 1	1	1	0	2
WD+VSL#3+OCA 2	2	2	2	6
WD+VSL#3+OCA 3	1	1	0	2
WD+VSL#3+OCA 4	2	1	1	4
WD+VSL#3+OCA 5	0	1	0	1
WD+VSL#3+OCA 6	1	1	0	2

WD+VSL#3+OCA 7	2	1	1	4
WD+VSL#3+OCA 8	1	1	0	2
WD+VSL#3+OCA 9	2	1	0	3
WD+VSL#3+OCA 10	2	1	1	4

Supplementary table S5. Summary of intestinal bile acid transport-, xenobiotic handling-, and drug metabolism-related gene expression.

Gene	Protein / Function	VSL#3+OCA vs OCA	Protein validation
Cyp2b10	Cytochrome P450 2B10; xenobiotic/drug-metabolizing enzyme, not a transporter	No clear numerical difference	No
Cdh17	Cadherin-17; intestinal epithelial adhesion molecule associated with intestinal absorptive epithelial phenotype	No clear numerical difference	No
Abcc2	Mrp2; apical efflux transporter	Numerically lower	Yes
Abcc3	Mrp3; basolateral efflux transporter	No clear numerical difference	No
Slco2b1	OATP2B1-related organic anion transporter; uptake transporter for bile acid-related and xenobiotic compounds	No clear numerical difference	No
Slc51b	OSTβ; basolateral BA efflux	Numerically lower	No
Slc51a	OSTα; basolateral BA efflux	No clear numerical difference	No
Slc10a2	ASBT; apical BA uptake	No clear numerical difference	No

Comparisons are presented descriptively between the WD+VSL#3+OCA and WD+OCA groups. Because treatment allocation for both VSL#3 and OCA was performed at the cage level and only two independently allocated cages were available per experimental arm, individual mice were not considered independent experimental replicates for between-group treatment comparisons. Accordingly, no inferential statistical testing was used to establish treatment-group differences.

Supplementary table S6. Number of animals used for each analysis.

Analysis	NC	WD	WD+OCA	WD+VSL#3	WD+VSL#3+OCA	Notes
Initial allocation	10	10	10	10	10	Final groups predefined at study initiation
Available after biopsy	10	10	9	8	10	One WD+OCA and two WD+VSL#3 mice died after biopsy
Histology/NAS	10	10	9	8	10	Endpoint liver sections
OGTT, pre-OCA	10	10	9	8	10	
BA analysis	3	4	5	6	8	Available qualified samples
PK analysis	-	-	8	-	7	OCA-treated groups only
Microbiome profiling, pre-OCA	10	16	-	18	-	Based on available fresh fecal samples
Microbiome profiling, endpoint	10	10	6	8	10	Based on available fresh fecal samples
Intestinal transporter qRT-PCR	10	10	9	8	10	Ileum tissue
Intestinal Mrp2 protein analysis	5	5	5	5	5	Available stained ileum sections

Analysis	NC	WD	WD+OCA	WD+VSL#3	WD+VSL#3+OCA	Notes
Sirius Red-positive area	10	10	9	8	10	Available stained liver sections
Oil red O-positive area	9	10	8	7	9	Insufficient or unavailable frozen liver sections
Hepatic triglyceride content	8	8	8	8	8	Insufficient remaining liver tissue in some animals
Inflammatory/fibrosis-related qPCR	9	9	9	8	8	Available liver RNA samples
Body weight, pre-OCA	10	10	10	10	10	Final predefined groups
Body weight (post)	10	10	9	8	10	Available post-biopsy animals
Food intake	2 cages	2 cages	2 cages	2 cages	2 cages	Cage was the experimental unit
Fluid intake	2 cages	2 cages	2 cages	2 cages	2 cages	Cage was the experimental unit

Supplementary Figures

Figure S1. Metabolic and histological profiles during Western diet feeding. Comparative analysis of (A) body weight, (B) food intake, and (C) liver to body weight ratio during the induction period between the NC and WD groups. (D) Blood glucose concentrations during the OGTT and the corresponding area under the curve (AUC) from 0 to 120 min after glucose administration. n = 10 for D. (E) Representative H&E-stained sections of the ileum and liver are also shown (×200). Data are presented descriptively as mean ± SD where applicable. Because treatment allocation was performed at the cage level and the number of independently allocated cages was limited, no inferential between-group statistical testing was used to establish treatment-group differences.

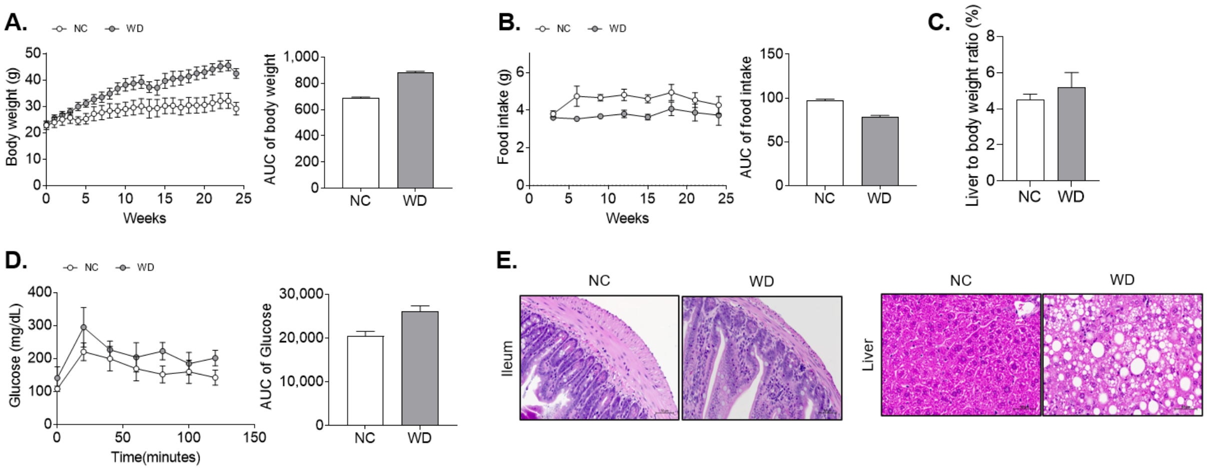

Figure S2. Effects of VSL#3 and OCA co-administration on food and fluid intake and hepatic inflammation- and fibrosis-related gene expression. (A) Food and fluid intake during the experimental period. Intake was measured at the cage level, normalized to the number of mice present in each cage, and expressed as estimated daily intake per mouse. Individual cage-level observations are presented descriptively. (B) Hepatic mRNA expression of the inflammation-related genes *Tnf-α* and *Ccl2*. (C) Hepatic mRNA expression of the fibrosis-related genes *Col1a1* and *Tgfb1*. Data are presented descriptively as individual values with the mean ± SD. For B–C, n = 9, 9, 9, 8, and 8 mice in the NC, WD, WD+OCA, WD+VSL#3, and WD+VSL#3+OCA groups, respectively. Because treatment allocation for both VSL#3 and OCA was performed at the cage level and only two independently allocated cages were available per experimental arm, individual mice were not considered independent experimental replicates for between-group treatment comparisons. Accordingly, no inferential statistical comparisons were used to establish treatment-group differences, and all observed patterns are presented as exploratory descriptive observations.

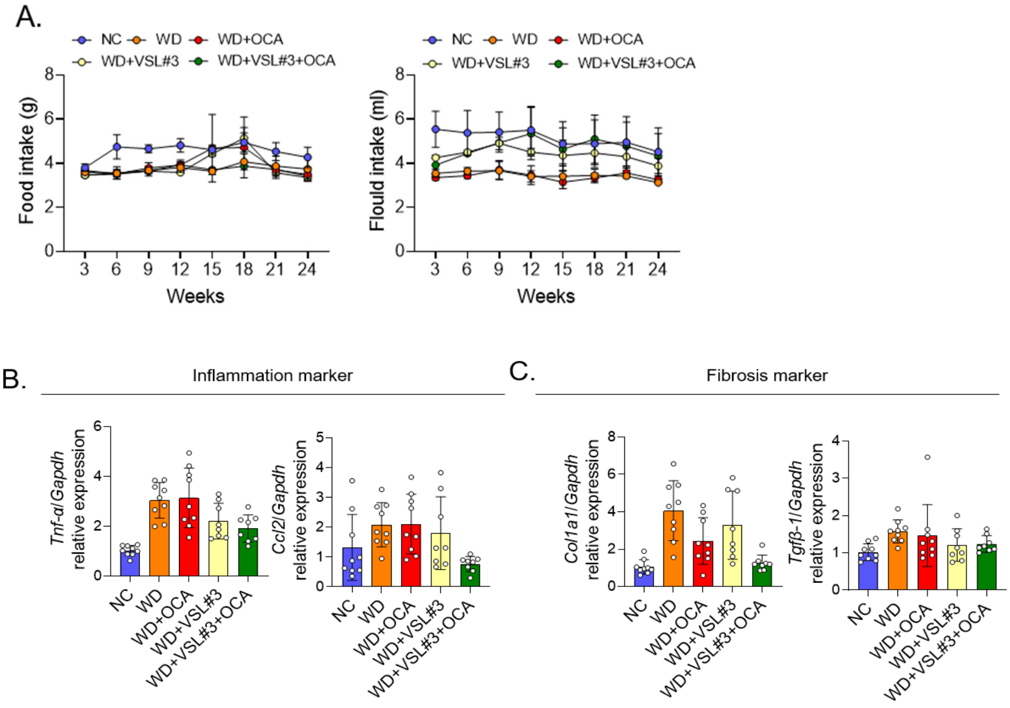

Figure S3. Serum biochemical parameters following VSL#3 and OCA co-administration in MASLD mice. Serum levels of (A) aspartate aminotransferase (AST), (B) total bilirubin, (C) high-density lipoprotein (HDL), and (D) low-density lipoprotein (LDL). Data are presented descriptively as individual values with the mean ± SD. n = 9, 9, 9, 7, and 9 mice in the NC, WD, WD+OCA, WD+VSL#3, and WD+VSL#3+OCA groups, respectively. Because treatment allocation for both VSL#3 and OCA was performed at the cage level and only two independently allocated cages were available per experimental arm, individual mice were not considered independent experimental replicates for between-group treatment comparisons. Accordingly, no inferential statistical comparisons were used to establish treatment-group differences, and the observed biochemical profiles are presented as exploratory descriptive observations.

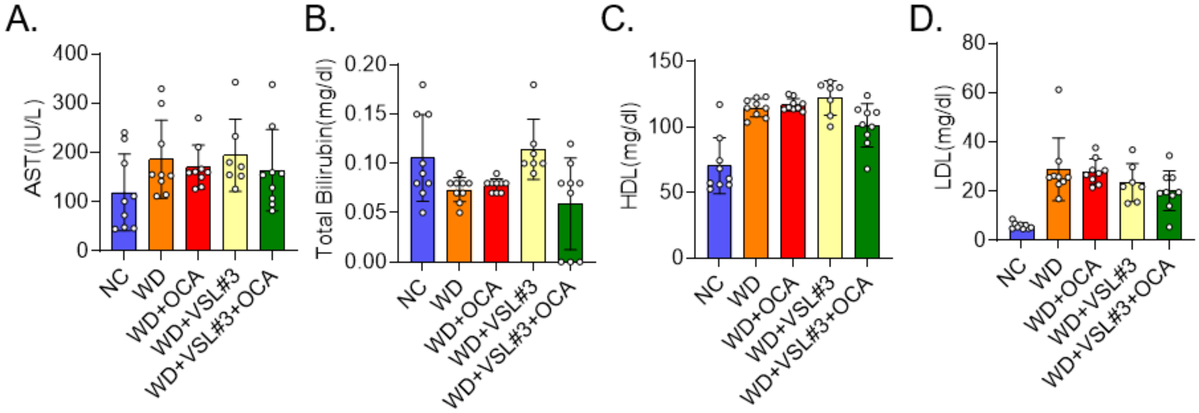

Figure S4. Hepatic expression of genes involved in bile acid synthesis and cholesterol handling following VSL#3 and OCA treatment. (A–C) Classical pathway gene expression and (D,E) alternative pathway gene expression in the liver. (F) Hepatic expression of genes related to cholesterol efflux and excretion. Data are presented descriptively as individual values with the mean ± SD where applicable. For A–F, n = 10, 10, 9, 8, and 10 mice in the NC, WD, WD+OCA, WD+VSL#3, and WD+VSL#3+OCA groups, respectively. Because treatment allocation for both VSL#3 and OCA was performed at the cage level and only two independently allocated cages were available per experimental arm, individual mice were not considered independent experimental replicates for between-group treatment comparisons. Accordingly, no inferential statistical comparisons were used to establish treatment-group differences, and all observed expression patterns are presented as exploratory descriptive observations.

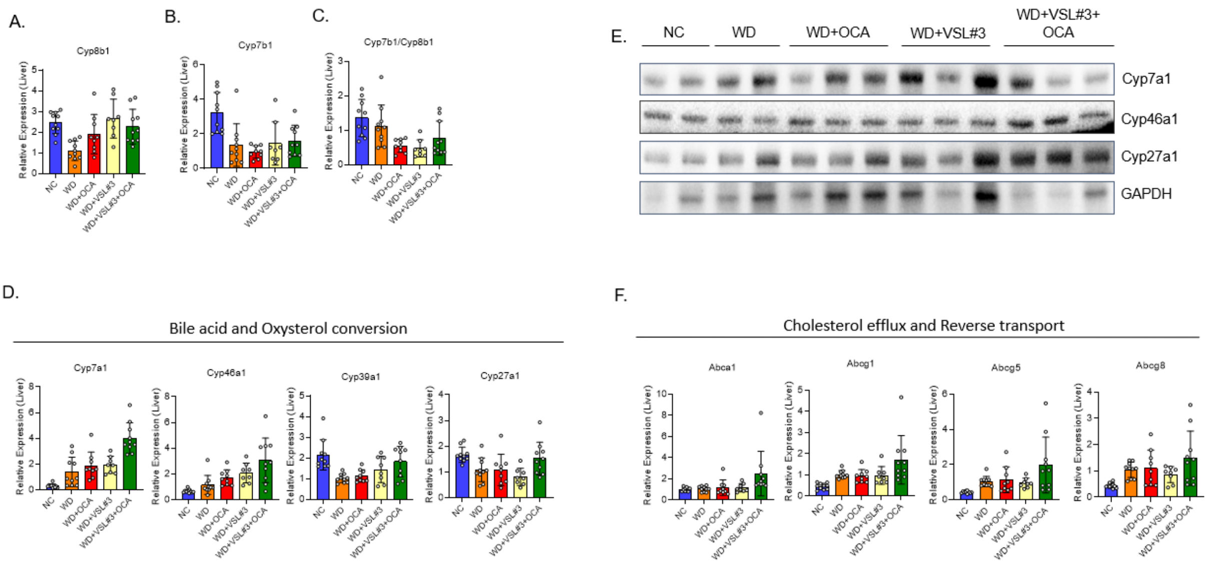

Figure S5. Associations between bile acid profiles and selected bacterial taxa. (A) Associations between unconjugated bile acids and bacterial taxa with reported bile salt hydrolase (BSH) potential. (B) Associations between taurine-conjugated BAs and *Lactobacillus johnsonii*. Associations were summarized using Spearman's rank correlation. Spearman's rank correlation coefficient (ρ) is reported descriptively without inferential p values. These analyses are exploratory and are not interpreted as confirmatory evidence of treatment-related effects.

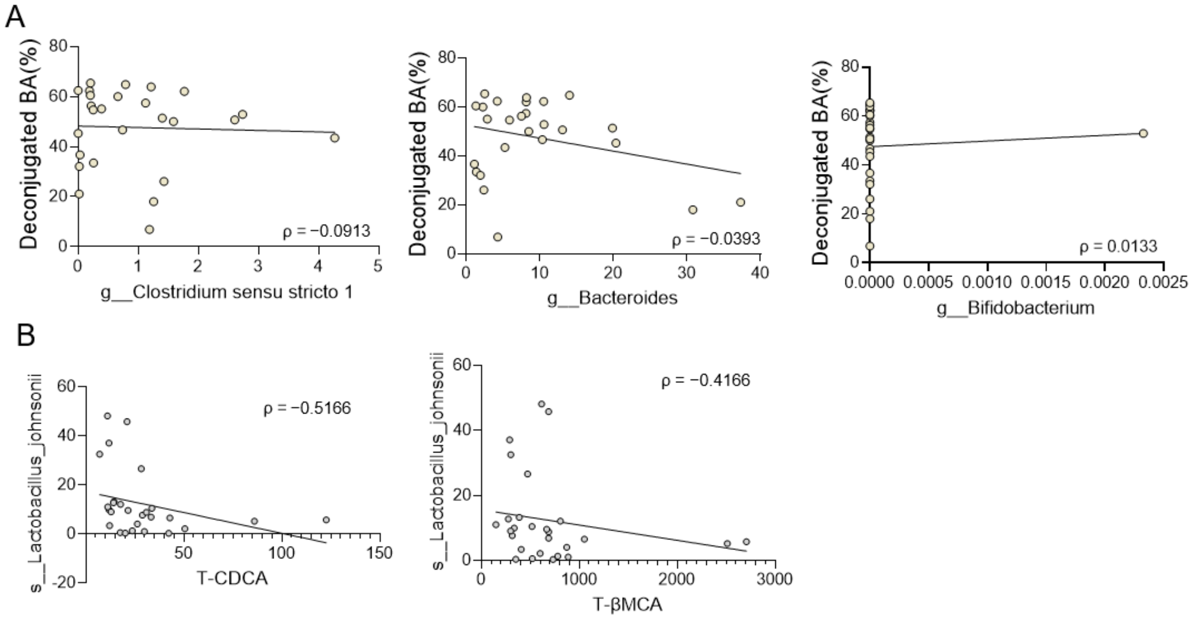

Figure S6. Intestinal transport-related gene expression, pharmacokinetic parameters, and exploratory associations between intestinal Abcc2/Mrp2 expression and selected bile acids in MASLD mice. (A) Intestinal expression of genes related to bile acid transport, drug absorption, efflux, and metabolism. Data are presented descriptively as individual values with the mean ± SD. (B) T_{max} and (C) elimination half-life (T1/2) of OCA. PK data are presented descriptively as individual biological observations with the mean ± SD. (D,E) Exploratory associations between intestinal Abcc2/Mrp2 expression and hyodeoxycholic acid (HDCA), hyocholic acid (HCA), and δ/α-muricholic acid (δ/α-MCA), summarized using Spearman's rank correlation coefficient (ρ). Correlation coefficients are reported descriptively without inferential p values. Because treatment allocation for both VSL#3 and OCA was performed at the cage level and only two independently allocated cages were available per experimental arm, no inferential between-group statistical testing was used to establish treatment-group differences. All expression, PK, and correlation findings are therefore presented as exploratory descriptive observations.

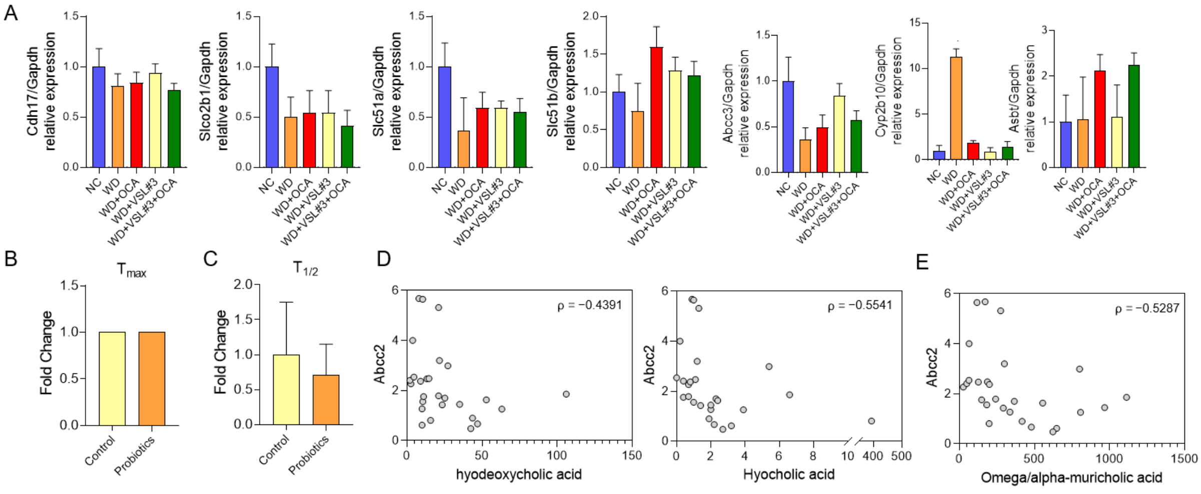

Supplement: Supplementary file 1 [file nutrients-18-03296-s001.zip › nutrients-4493596-supplementary.pdf]
